# Gene regulatory network inference with popInfer reveals the dynamic regulation of hematopoietic stem cell quiescence

**DOI:** 10.1016/j.isci.2025.114010

**Published:** 2025-11-11

**Authors:** Megan K. Rommelfanger, Marthe Behrends, Yulin Chen, Jonathan Martinez, Nikith Kurella, Nino Geisler, Deepthi Guturu, Martin Bens, Lingyun Xiong, Zijin Xiang, K. Lenhard Rudolph, Adam L. MacLean

**Affiliations:** 1 Department of Quantitative and Computational Biology, University of Southern California, Los Angeles, CA 90089, USA; 2 Research Group on Stem Cell and Metabolism Aging, Leibniz Institute on Aging, Fritz Lipmann Institute (FLI), Jena, Germany; 3 Core Facility Next Generation Sequencing, Leibniz Institute on Aging, Fritz Lipmann Institute (FLI), Jena, Germany; 4 Department of Stem Cell Biology and Regenerative Medicine, Broad-CIRM Center, Keck School of Medicine, University of Southern California, Los Angeles, CA 90089, USA; 5 Medical Faculty, Jena University Hospital, Friedrich Schiller University, Jena, Germany

**Keywords:** molecular physiology, gene network, cell biology

## Abstract

Inference of gene regulatory networks (GRNs) can reveal cell state transitions from single-cell genomics data. However, obstacles to temporal inference from snapshot data are difficult to overcome. Single-nuclei multiomic data offer a means to bridge this gap and derive temporal information using joint measurements of gene expression and chromatin accessibility in the same single cells. We developed popInfer to infer networks that characterize lineage-specific dynamic cell state transitions from joint gene expression and chromatin accessibility data. Benchmarking against alternative methods for GRN inference, we showed that popInfer achieves higher accuracy in the GRNs inferred. popInfer was applied to study single-cell multiomics data characterizing hematopoietic stem cells (HSCs) and the transition from HSC to a multipotent progenitor cell state during murine hematopoiesis across age and dietary conditions. From the networks predicted by popInfer, we discovered gene interactions controlling entry to/exit from HSC quiescence that are perturbed in response to diet or aging.

## Highlights

• PopInfer integrates multiomic data to infer GRNs that control dynamic cell state transitions
• Distinct from alternatives, popInfer fits a model in the joint space: RNA-seq + ATAC-seq
• PopInfer discovers hematopoietic stem cell networks perturbed by diet and age
• PopInfer reveals that mutual inhibition between Mecom and Cdk6 controls stem cell quiescence

## Graphical abstract

**Figure:**
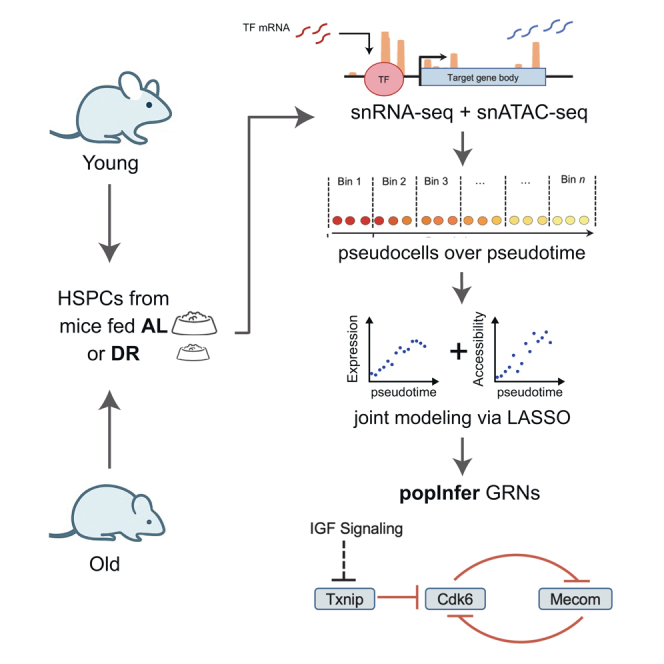

## Teaser

Molecular biology; Transcriptomics; Biocomputational method; Molecular mechanism of gene regulation

## Introduction

Hematopoietic stem cells (HSCs) maintain the blood system throughout life. Mammalian hematopoiesis occurs primarily in the bone marrow, where HSCs, residing in restricted niches,^1^^,^^2^^,^^3^ give rise to multipotent progenitor cells, and subsequent lineage-restricted progenitor cell populations of various identities.^4^^,^^5^ This process is tightly controlled by a range of cell-intrinsic and extrinsic factors to avoid the aberrant proliferation of stem/multipotent cells. Regulatory mechanisms controlling the lineage commitment steps during hematopoiesis are mediated primarily by gene regulatory networks (GRNs). The best-studied example of such a GRN is the mutually inhibiting transcription factor pair (*GATA1* and *PU.1*) that controls erythroid vs. myeloid (granulocyte/monocyte) lineage commitment.^6^ Another example, mutual inhibition between *IRF8* and *GFI1*, controls lineage commitment within myeloid (granulocyte vs. monocyte) lineages.^7^ Larger GRNs can also be constructed and have identified mechanisms that regulate cell state stability.^8^ However, hematopoiesis involves many other cell fate decisions—from lineage commitment to entry into/exit from quiescence—for which the GRN interactions responsible are incomplete or unknown. Importantly, these include the early cell fate decision during hematopoiesis whereby HSCs lose their self-renewal/stemness and transition to a non-stem multipotent progenitor cell fate.^9^ A combination of transcriptional and epigenetic factors is implicated in the loss-of-stemness transition from HSCs to multipotent progenitors.^10^^,^^11^

Given their required longevity,^12^^,^^13^ HSCs seldom divide, and are primarily kept in a non-proliferating/quiescent state to protect against replication-induced damage.^13^ Rounds of HSC division––and thus cellular aging––are associated with reduced regenerative potential, reduced lymphopoiesis (myeloid skewing), and decreased clonal diversity.^14^ These age-associated effects lead to an increase in the total number of HSCs and an altered composition of the peripheral blood.^13^^,^^15^^,^^16^ The age-related increase in HSC numbers is driven by the expansion of myeloid-biased HSCs, giving rise to disproportionately myeloid progeny.^13^^,^^16^^,^^17^ This skewing, caused in part by DNA damage and its downstream effects,^18^ can be ameliorated by depleting myeloid-biased HSCs.^19^ Epigenetic remodeling and metabolic changes also contribute to impaired HSC function with age.^20^

Mild dietary restriction (DR), which typically consists of 60–80% of the *ad libitum* food intake by weight, has been established as a highly geroprotective intervention, leading to increases in lifespan between 20 and 30% in mice, attenuation of aging-associated inflammation, and delayed onset of cancer and frailty.^21^^,^^22^^,^^23^ We have previously shown that DR in young to middle aged mice (early aging) attenuates aging-related increases in HSC numbers, and improves HSC repopulation capacity, potentially through better maintenance of stem cell quiescence.^16^ The transcriptional and metabolic changes that DR induce remain largely unknown, as does the specific mechanisms by which DR alleviates aging phenotypes in HSCs. The identification of GRNs controlling cell fate decisions in hematopoiesis that are regulated through aging and/or DR could lead to an understanding of how aging drives aberrant hematopoiesis and how it may be influenced by DR.

Gene regulatory network inference seeks to determine networks of gene-gene interactions from data. Building on methods to infer GRNs from gene expression data in bulk samples (RNA-seq),^24^^,^^25^ new methods to infer gene networks have been developed in light of the higher resolution obtained by single-cell RNA-sequencing (scRNA-seq). These are built on the premise that the higher-resolution offered by decomposing bulk samples into single cells can improve GRN inference, and that the single-cell noise does not overwhelm the signal. These include methods rooted in statistical learning,^26^ dynamical systems theory,^27^^,^^28^ tree-based approaches,^29^ information theory,^30^^,^^31^^,^^32^ and time series analysis.^33^^,^^34^ More recently, methods also consider dynamic changes to the network topology itself.^35^ Methods have also been introduced that make use of chromatin accessibility in addition to gene expression.^36^^,^^37^^,^^38^^,^^39^^,^^40^^,^^41^ As a result of the variety of underlying models, these methods produce differing networks, and thus, benchmarks have sought to compare their performance on both real and simulated data.^42^^,^^43^^,^^44^ Despite their promise, it has been challenging to achieve high performance on GRN inference with single-cell data overall,^45^ especially temporally ordered data^34^; indeed, it can be hard to outperform linear methods.^46^ Moving beyond static networks represents another key challenge^47^; in some cases, the performance of pseudotime-based methods is worse with the true ordering than with randomly ordered cells.^33^

Predicting gene regulatory interactions controlling processes during development or stem cell differentiation has certainly improved as the resolution of the data increases with single-cell technologies. Nevertheless, theoretical limits exist, both in our ability to infer dynamics from snapshot gene expression data^48^ and in our ability to control error rates.^49^ New computational and measurement technologies may be able to (at least in part) overcome these challenges.^50^^,^^51^^,^^52^^,^^53^ Recent advances have enabled the joint measurement of gene expression by RNA-sequencing (RNA-seq) and chromatin accessibility by assay for transposase-accessible chromatin by sequencing (ATAC-seq) in the same single cells^54^^,^^55^ and patterns of variability therein,^56^ offering new potential to learn complex dynamic gene regulatory processes.

Here we present popInfer: GRN inference with pseudocells over pseudotime, a new method to infer GRNs using joint single-cell multiomic data. popInfer learns directed signed GRNs. That is, popInfer can distinguish not only the direction of the interaction (regulator gene to target gene) but also its sign (activating vs. inhibitory). We tested popInfer on unperturbed hematopoiesis and on systems exposed to dietary restriction and/or aging, focusing on the dynamics of the transition from stem cells to multipotent progenitors. Through comparison with reference data gathered by chromatin immunoprecipitation assay with sequencing (ChIP-seq), we demonstrated that popInfer outperforms alternative GRN inference methods that run on scRNA-seq data alone. We show that this performance gain is in part derived from our pseudotime model: performance drops when cells are randomly ordered. popInfer also outperforms alternative multiomic GRN inference methods. In some cases, an over-reliance on literature-informed network interactions by alternative approaches leads to inferred networks comprised of TFs with no known connection to hematopoiesis.

Using popInfer, we predicted GRNs controlling the transition from HSCs to multipotent cells under various conditions. Comparative analysis of the networks revealed a core GRN governing HSC quiescence by the mutual inhibition of *Mecom* and *Cdk6*. We also identified a direct association between IGF signaling and *Mecom-Cdk6* dynamics. An increased level of quiescence is observed in young HSCs with DR. Our results show how HSC quiescence is controlled by IGF signaling-mediated changes in young hematopoiesis by *Mecom-Cdk6* and how this regulation wanes as IGF signaling decreases with age. We analyze the inferred GRN by assessment against three additional datasets (two in mice and one in human),^57^^,^^58^^,^^59^ which provide strong evidence to corroborate the predictions by recovering the same expression patterns across various perturbed states of mouse and human HSCs.

## Results

### The stem-to-multipotent hematopoietic transition is maintained throughout lifetime and dietary perturbations

**Figure 1:**
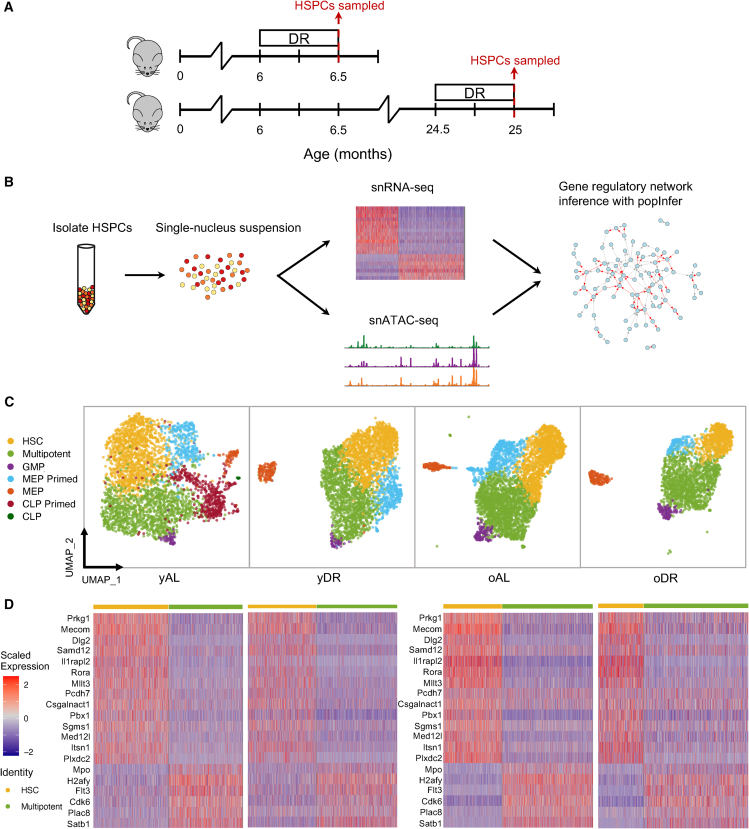
*Joint multiomic data characterize hematopoiesis across diet and age* (A) Overview of experimental design: mice undergo diet restriction (DR) at a young or old age prior to sampling of hematopoietic stem and progenitor cells (HSPCs). (B) HSPCs are sorted from the bone marrow and isolated for single nuclear (sn) RNA and ATAC sequencing, and re-integrated into a gene regulatory network inference model: popInfer. (C) HSPCs for each experimental condition are clustered with cluster annotations made using hematopoietic marker genes. GMP, granulocyte-monocyte progenitor; MEP, megakaryocyte-erythroid progenitor; CLP, common lymphoid progenitor. (D) Heatmaps of differentially expressed genes (top 20 ranked by log-fold change) between HSCs and multipotent progenitors; heatmaps correspond to samples above in (C). yAL, young *ad libitum*; yDR, young DR; oAL, old *ad libitum*; oDR, old DR.

We studied early hematopoiesis in young and old mice and their response to DR via joint multiomics: concurrent sequencing of single-nucleus gene expression (snRNA-seq) and chromatin accessibility (snATAC-seq) using the 10X multiome platform (Figure 1A). Data were generated from young (∼6 months) and old (∼24 months) mice. Mice were either fed *ad libitum* continually until bone marrow isolation at young (yAL) or old age (oAL) or underwent mild dietary restriction (DR; 30% reduction in food intake by weight) for the two weeks prior to bone marrow isolation at young (yDR) or old age (oDR) to investigate early response to nutrient deprivation. Bone marrow cells were isolated, and Lineage ^-^ Sca-1^+^ cKit ^+^ (LSK) cells were sorted and used for nuclei isolation and subsequent single-nucleus joint multiome sequencing (Figure 1B). RNA-seq and ATAC-seq datasets were preprocessed (see STAR Methods), and each modality was analyzed and then integrated for gene regulatory network inference and analysis.

Aging and diet both affect the rates of cell division in the HSC pool, thereby influencing the self-renewal and quiescence propensities of stem cells. These altered phenotypes in the HSC pool can affect hematopoiesis overall and manifest through different frequencies of mature blood cells in the bone marrow and peripheral blood. The earliest cell fate decisions during hematopoiesis—whereby HSCs may lose their self-renewal capacity during asymmetric or symmetric cell divisions when transiting to multipotent progenitor cells (MPPs)—exert influence over every cell fate decision that follows. We thus investigated how hematopoietic subpopulations change during early stages of differentiation. Joint multiomic data provide a unique opportunity to identify how these transitions are transcriptionally regulated.

Unsupervised clustering via the Louvain algorithm (see STAR Methods) identified 5–7 clusters in each condition. Via known marker genes from HSC, multipotent, and lineage-restricted early progenitor populations, cell state identities were assigned to each cluster (Figures 1C and S1). Across all conditions, the majority of cells belong to HSC/multipotent clusters. We also identified lineage-primed and lineage-restricted cells for each condition, including megakaryocyte-erythroid progenitors (MEPs), granulocyte-monocyte progenitors (GMPs), and common lymphoid progenitors (CLPs) (Figure 1C). Of note, CLPs were only identified in yAL. CLPs are known to decline with age; these data also support our previous studies of DR that found it suppresses the generation of CLPs in young mice.^16^

We performed differential gene expression analysis and studied the stemness and multipotent gene expression signatures present across age and dietary conditions. We analyzed the top differentially expressed genes between HSCs and multipotent cells in yAL (ranked by log2 fold change) across samples in (Figure 1D). Known marker genes for HSCs emerged, including *Mecom* and *Mllt3*; in the multipotent cluster multipotent marker genes were present, including *Flt3* and *Mpo*.^7^^,^^60^ Analysis of the pattern of HSC/multipotent differential expression across all samples revealed a core hematopoietic differential gene expression pattern across all conditions. Notably, despite the relative homogeneity of the HSC/multipotent progenitor cell pool and the clear changes that HSCs undergo throughout life, the transcriptional signature distinguishing HSCs from multipotent progenitor cells is clearly maintained throughout life and dietary restriction.

### Concurrent measurement of gene expression and chromatin accessibility enables inference of dynamic gene regulatory interactions

GRN inference methods that seek to infer gene regulatory interactions fall under two categories: time-dependent and time-agnostic methods. Several time-dependent GRN inference methods have been developed specifically for application to non-temporal (“snapshot”) data. These infer the temporal process from a population of heterogeneous single cells via trajectory inference/pseudotime.^27^^,^^30^^,^^33^^,^^61^^,^^62^ The non-discrete nature of cell states during hematopoiesis strengthens the case for continuous pseudotime-based approaches.^63^ However, whether pseudotime values for individual genes contain useful dynamical information is highly dataset-dependent and remains an open question. Deshpande et al.^33^ showed that interpreting pseudotime values explicitly did not improve inference versus considering only the order of cells along pseudotime. Moreover, GRN inference with pseudotime-ordered cells was not necessarily better than with randomly ordered cells. Ventre et al.^34^ also showed that the performance of the pseudotime-based GRN inference approaches was low (close to random).

**Figure 2:**
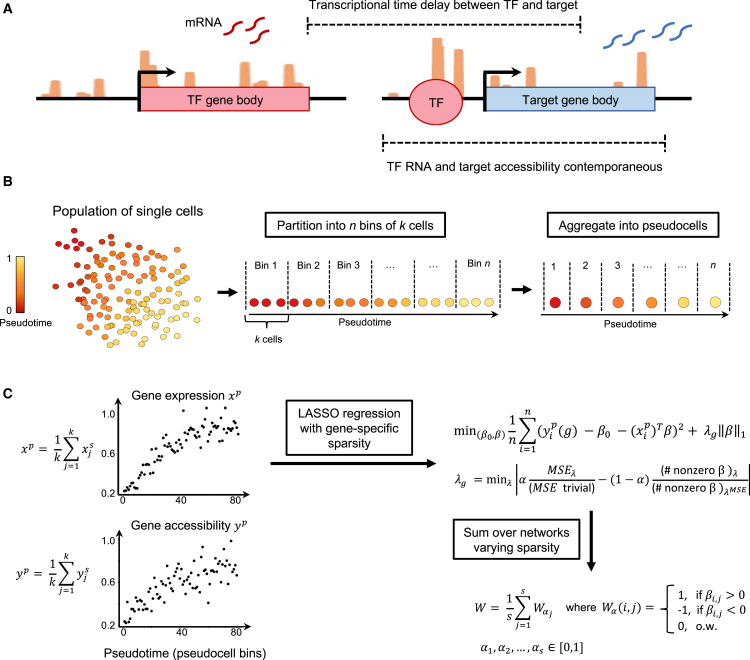
*popInfer: gene regulatory network inference with multiomic data* (A) Schematic depicting transcription factor (TF) gene expression and its regulation of a target gene. TF transcription is followed by a time delay before TF regulation can be detected in the target gene expression. TF expression is more concurrent with the accessibility of the target gene. (B) Overview of data processing for popInfer. Cells are ordered over pseudotime and binned into *n* equally sized bins of *k* cells. (C) Overview of popInfer workflow. Cells belonging to a bin are aggregated to form a pseudocell, where $x_{j}^{p}$ is the single-cell expression of the *j*th cell in a pseudocell bin and *x*^*p*^ is the pseudocell gene expression. $y_{j}^{s}$ is the gene accessibility score of the *j*th cell in a pseudocell bin, and *y*^*p*^ is pseudocell gene accessibility. A LASSO regression model is run on the *n* pseudocells, where pseudocell accessibility *y*^*p*^ is predicted from pseudocell gene expression *x*^*p*^. The selection of the regularization term *λ*_*g*_ is gene-specific, governed by a tradeoff between sparsity and the mean squared error (MSE); *α*∈[0,1]. MSE_*λ*_ is the MSE of the LASSO model for a given *λ* value and “MSE trivial” is the MSE for a trivial model with *β* = 0. The number of nonzero *β* coefficients is calculated for the LASSO model for a given *λ*, and for the LASSO model that achieves the optimal MSE (*λ*^MSE^).

A motivating factor in the use of pseudotime for temporal inference is the expected time lag between transcriptional changes in the regulator and those in the target (Figure 2A). By assigning pseudotime values to cells, time lags can be modeled, even for “snapshot” data with no explicit measurement of the dynamics. Key to implementing time-series-based methods is data comprising cells that are equally spaced in time (or interpolating time-equidistant cells). That is, a reliance on the explicit use of pseudotime values in the model, which, as discussed above, is an assumption that stands on shaky ground. However, inference without a temporal/pseudotemporal measure precludes the investigation of dynamic relationships between regulator genes and their targets, impeding attempts to address causality without additional measurements.

Joint multiomics offers more contemporaneous measurements of interacting genes, regulators, and targets, via the gene expression of the transcription factor (regulator gene) and the chromatin accessibility of the target. popInfer leverages these data to fit a regression model to predict the chromatin accessibility of the target gene from the gene expression of the regulator (Figure 2A). While there are certainly caveats to this approach, e.g., the different measurement technologies employed, these values occur more contemporaneously than regulator expression and target gene expression. Thus, a core assumption of popInfer is that if a TF regulates a given target, there should be a relationship between the pseudotemporal gene expression profile of the TF and the pseudotemporal chromatin accessibility of the target gene. Through this use of coupled assays, popInfer infers directional interactions that can be used to generate causal hypotheses; however, additional evidence will be required to prove their necessity. We note as an aside that the GRN inference literature is rife with claims of causality, which—we would argue—are often overblown.

Single-nuclei RNA-seq and ATAC-seq data are sparse measurements in which many per-cell transcripts or peaks will be missed during sampling. This motivates our construction of pseudocells: bins of several single nuclei measurements for a given gene or ATAC-seq peak. The construction of pseudocells required means by which to partition cells into groups that were transcriptionally similar along the axis of interest (in this case, stem cell differentiation). This may constitute an appropriate opportunity to use pseudotime as a means to order cells by their similarity along a trajectory, i.e., avoiding the need to interpret pseudotime values explicitly. Thus, we used pseudotime to order cells along a trajectory as input to partitioning them into bins. Bins of cells were used to construct pseudocells, which are the input data to GRN inference by popInfer (Figure 2B). Pseudocell gene expression (*x*_*p*_) is defined as the average expression of the cells in that bin. The gene accessibility score is derived from the snATAC-seq data, taking into account peaks in the gene body and its promoter region via ArchR’s GeneScoreMatrix gene accessibility scores. This score takes into account peaks that lie in the gene body and the promoter region (5 kb upstream of the TSS). Note that this score does not take into account enhancer information (which can be gained from ATAC-seq) nor methylation marks that can distinguish euchromatin from heterochromatin and thus identify poised loci (which cannot be resolved from ATAC-seq but can from other sequencing approaches). Pseudocell gene expression (*x*_*p*_) and gene accessibility (*y*_*p*_) scores are then defined to be the average of these values for the cells in the corresponding bin (Figure 2C).

By constructing pseudocells with expression and gene accessibility scores, popInfer seeks to infer a network that describes the dynamics of the cell state transition that is captured by these coupled trajectories. popInfer assumes a static GRN: i.e., it is built on the premise that by considering the information contained in the trajectories that capture a dynamic transition, regulations controlling this transition can be inferred. Alternative approaches, e.g., considering dynamic networks^41^ may provide orthogonal information but may also lose power by not considering at once the entire trajectories. popInfer performs LASSO regression^64^ using pseudocell gene expression values to predict pseudocell gene accessibility scores (Figures 2B and 2C). For each target gene (*g*), gene-specific sparsity (*λ*_*g*_) is determined via a tradeoff between model sparsity and mean-squared error (MSE). This tradeoff is controlled by a parameter *α*∈[0,1], which is fixed for all genes. When *α* = 0, *λ*_*g*_ is selected such that we obtain a trivial model whereby *β* = 0. When *α* = 1, *λ*_*g*_ is selected such that we obtain a model with optimal MSE.

For a given *α*, the LASSO outputs a matrix of inferred coefficients *β*. These values are binarized, with their sign maintained, to obtain a network matrix *W*_*α*_ (Figure 2C). Preserving the sign of the LASSO coefficients allows popInfer to learn information regarding activation versus inhibition for interacting genes. The final output of popInfer is *W*, the averaged sum of the *W*_*α*_ for a sequence of *α* values in the range [0,1]. Choosing *α* closer to zero produces sparser networks; larger values of *α* produce denser networks. In this study, we explored the sequence of *α* values {0,0.001,0.002, …,0.4}. As a result, most gene-gene pairs are assigned weights of 0. While this has the potential to inflate the false negative rate, we believe this to be overall advantageous because it is often unclear how to assign weight thresholds to select a network during GRN inference. popInfer assigned edges with zero weight are automatically ruled out. Subsequently, a weight threshold >0 can be set if desirable to select networks with the highest confidence edges.

### popInfer outperforms gene regulatory networks inference methods based only on gene expression

To evaluate the performance of our proposed methods, we studied the results of networks inferred by popInfer and compared these with other methods for GRN inference from single-cell gene expression data. As a point of comparison, we used reference ChIP-seq data from ChIP-atlas, as defined by the benchmarking framework BEELINE.^43^ For the inference methods to which we compare popInfer, we selected GENIE3,^24^ PIDC,^31^ TENET,^30^ and SINCERITIES.^61^ These methods have all shown strong performance under different conditions and are built upon diverse mathematical frameworks. Benchmarking methods are inherently difficult given the challenge of identifying cell state-specific true positive interactions. Here, we use ChIP-seq performed on hematopoietic cells from whole bone marrow, which offers a set of true interactions, but since we are looking at a narrow subset of bone marrow cells and a small set of genes, we can expect to recover at best only a fraction of all possible true positive interactions in the reference. It is, however, informative to compare the relative performance of methods at recovering the highest weighted interactions from the reference dataset.

We began by studying the HSC to multipotent transition in the yAL sample. As input to GRN inference methods, we used the same set of 104 genes that were identified as differentially expressed between HSCs and multipotent progenitors (see STAR Methods). For evaluation, we compare the results of each method via the area under the early precision-recall curve (AUEPRC). For a specified range of recall values, the AUEPRC is the area under the precision recall curve for the specified range of recall values, normalized by the total recall range. We focused on early precision-recall since we expect true networks to be sparse relative to the number of possible interactions. Under these conditions, the AUEPRC is well-suited to assess method performance, specifically to assess whether methods perform well on their highest-ranked network interactions. For the HSC to multipotent transition in yAL, we evaluate AUEPRC over recall values in the range [0,0.1].

**Figure 3:**
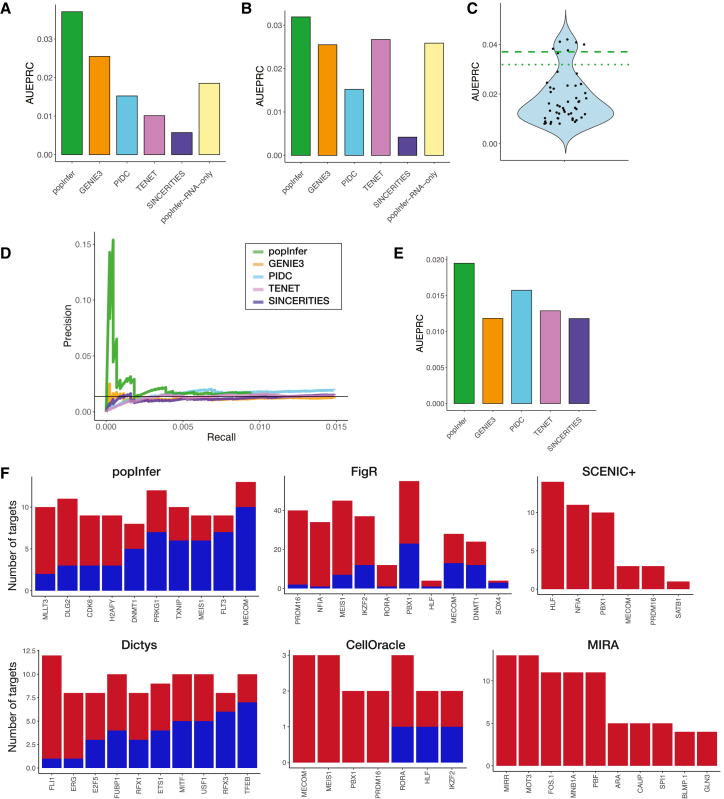
*popInfer benchmark comparison with alternative GRN inference methods* (A) Network inference evaluation of the HSC to multipotent transition in yAL for cell cycling pseudotime. Bar plots give the area under the early precision-recall curve (AUEPRC) for recall values in the range [0.0.1]. Networks were compared against a set of true positive gene interactions defined by ChIP-Atlas. (B) Network inference evaluation with AUEPRC as for (A), with cell cycle effects regressed out. (C) Violin plot of the area under the early precision-recall curve for yAL for popInfer, using pseudocells that were comprised of single cells randomly sampled from cell-type clusters (as opposed to pseudotime-ordered). Green dashed line: popInfer AUEPRC from (A); green dotted line: popInfer AUEPRC from (B). (D) Benchmarking the HSC to GMP transition in oAL; true positives from ChIP-Atlas. Early precision-recall curves for each method are shown. (E) Bar plot of AUEPRC for the curves in (D); recall range [0,0.015] for the HSC to GMP transition in oAL. (F) Comparison of the top regulator genes predicted by each of six multiomic GRN inference methods for the HSC → MPP transition. Blue: inhibitory; red: activating (or no sign given).

In yAL, we ran inference twice on two different sets of pseudotime values: one that was computed on expression counts that included cell cycle effects, and one that was computed on cell cycle regressed expression counts (see next section for full justification). We found that popInfer outperforms the other methods in both the cell cycle (Figure 3A) and cell cycle regressed (Figure 3B) iterations of inference on the yAL data. We also tested how popInfer performs when run on RNA data alone (i.e., predicting gene expression values from gene expression, as opposed than predicting gene accessibility values from gene expression; “popInfer-RNA-only”). In agreement with our logic in constructing the model (Figure 2A), we observed a decrease in performance when using the RNA-only model, in keeping with the assumption that the time delay between TF expression and target gene transcription incurs a cost. We found that popInfer-RNA-only performed similarly to GRN inference methods relying on scRNA-seq data alone (Figures 3A and 3B).

Next, we challenged our assumption that pseudotime was necessary for the construction of pseudocells as input to popInfer. We constructed new sets of pseudocells by randomly sampling cells from within each cluster (either HSC or multipotent) to create pseudocells. Figure 3C shows the AUEPRC of popInfer applied to these cell-type specific random pseudocells for 50 replicates. The dashed and dotted green lines are the AUEPRC value of popInfer from Figures 3A and 3B, respectively, showing that constructing pseudocells using pseudotime improves results over using cell-type resolution to create pseudocells.

Since relatively few genes comprised the set that were differentially expressed along the HSC to multipotent transition (a total of 111 TF-target pairs in the reference ChIP-seq data), we also studied a hematopoietic trajectory described by a larger set of genes: differentiation from HSCs to granulocyte-monocyte progenitors (GMPs). We selected those cells belonging to HSC, intermediate multipotent, and GMP populations and studied these hematopoietic fate decisions in oAL. For features, we selected the set of differentially expressed genes between the HSC and GMP clusters in the oAL dataset, giving us 565 genes. As a result of the larger set of input genes, there were 4374 TF-target gene pairs included in the reference. We chose to use the cell cycle regressed pseudotime values for this branch of differentiation in order that cell cycle effects did not dominate over subtler gene expression/accessibility differences from the relatively few cells in the GMP cluster.

Tested on the HSC-to-GMP differentiation trajectory, popInfer outperforms other methods (Figures 3D and 3E). In the early precision-recall curve, the precision values of popInfer are large relative to the other methods for small recall values, meaning that popInfer performs well at the early detection of true positives (Figure 3D). This behavior was conserved when tested with different inputs: number of pseudocells and *α* value sequences (Figure S2).

### popInfer outperforms multiomic gene regulatory networks inference methods for learning cell type-specific networks

We benchmarked the performance of popInfer against other GRN inference methods that employ multiomics to infer regulatory networks. popInfer was compared to five alternative multiomic GRN inference methods: SCENIC+,^40^ CellOracle,^36^ Dictys,^41^ FigR,^65^ and MIRA.^66^

Multiomic methods for GRN inference utilize a wide range of models that often rely on multiple steps of filtering either the data or the literature to construct base networks (e.g., informed by chromatin accessibility and known TF binding sites), which are then further refined by various methods to produce a final network. These filtering steps result in different sets of features comprising the inferred GRNs across the tools. It is thus difficult to compare TF—target gene relationships using performance metrics such as AUPRC, as it requires a consistent feature set for all methods. Hence, we consider alternative approaches to assess the quality of the hematopoietic networks inferred by each method, taking into account both general network features and supervised analysis of known HSC networks.

For the HSC-to-multipotent transition, we inferred networks based on the set of 104 genes that were identified as differentially expressed between HSCs and multipotent progenitors. Networks were pruned to the same number of edges as popInfer’s network (if a network contained fewer edges than popInfer’s network, all edges were included). Comparison of the inferred networks in terms of conserved edges showed little overlap (Figure S3A), mostly due to the small sets of mostly non-overlapping regulators (Figure S3B). Notably, the networks inferred by SCENIC+ and CellOracle contained few edges overall. Among the target genes in these networks, there is more overlap seen overall (Figure S3C). Notably, popInfer target genes overlap with alternative methods more than any other approach. The low level of edge overlap overall highlights the impact of varied approaches to feature inclusion/exclusion and the need for alternative approaches for network assessment and comparison. Genes were ranked by their number of outgoing edges and whether they encode for transcription factors that are predicted to activate or inhibit each target. We compared the methods in terms of the top predicted transcription factors with the greatest number of targets (Figure 3F). In cases where the GRN inference method predicts the sign of interaction (all multiomic methods tested except MIRA and SCENIC+), this information is also included. The top 10 TFs according to the total number of inferred targets are plotted (Figure 3F). Some methods significantly prune the TFs considered, and therefore, evaluate fewer than 10 TFs.

In terms of the proportion of activating to inhibitory interactions among those predicted (excluding MIRA and SCENIC+, which predicted none), Dictys predicted approximately equal numbers of activating and inhibitory interactions, similarly to popInfer. CellOracle and FigR each predicted a few inhibitory interactions but were dominated by activating TF—target gene interactions. Differences between methods in terms of cell type specificity were also evident: while most top TFs belonged to families with crucial functions for or known associations with HSC maintenance (e.g., MLLT3, MEIS1), Dictys and MIRA predicted several TFs with no known roles in hematopoiesis, some of which belonged to TF families involved in ubiquitous cellular machinery, such as the E2F TF family. In contrast, Dictys was the only method to identify a top regulator of the HSC → MPP transition (Figure 3) that overlapped with an analysis of regulators of human hematopoiesis in response to MECOM perturbation (mimicking a haploinsufficiency syndrome)^67^: the E26 transformation-specific (ETS) family.

### popInfer identifies a gene regulatory network controlling the hematopoietic stem cell-to-multipotent transition via insulin-like growth factor signaling

To study the impact of aging and dietary restrictions on the HSC-to-multipotent cell fate transition, we ran popInfer on a set of 104 genes differentially expressed between HSCs and multipotent cells in at least one experimental condition. Gene interactions predicted by popInfer were defined as those with GRN edge weights >0.4. We considered two scenarios: networks constructed via the inclusion or the exclusion of cell cycle effects. The cell cycle exerts important effects on the transcriptional state of the cell and subsequent gene regulation^68^^,^^69^ (Figure S4), leading to cell cycle effects that can dominate in pseudotime. In such cases, performing cell cycle regression can prevent these transcriptional signatures from obscuring other regulatory processes. However, it is not possible to entirely separate the stem cell state from the cell cycle status. We thus considered GRNs inferred under both conditions of using the unregressed, cell cycling pseudotime or using cell cycle-regressed (non-cycling) pseudotime. In the former, no cell cycle regression is performed, and cell cycle effects can be observed across cell populations (Figures S1C and S4); in the latter, cell cycle effects are regressed out of the snRNA-seq data before pseudotime assignment (Figure S1D). In both cases, diffusion pseudotime is used to construct a pseudotemporal ordering of cells (Figures S1C and S1E).^70^

**Figure 4:**
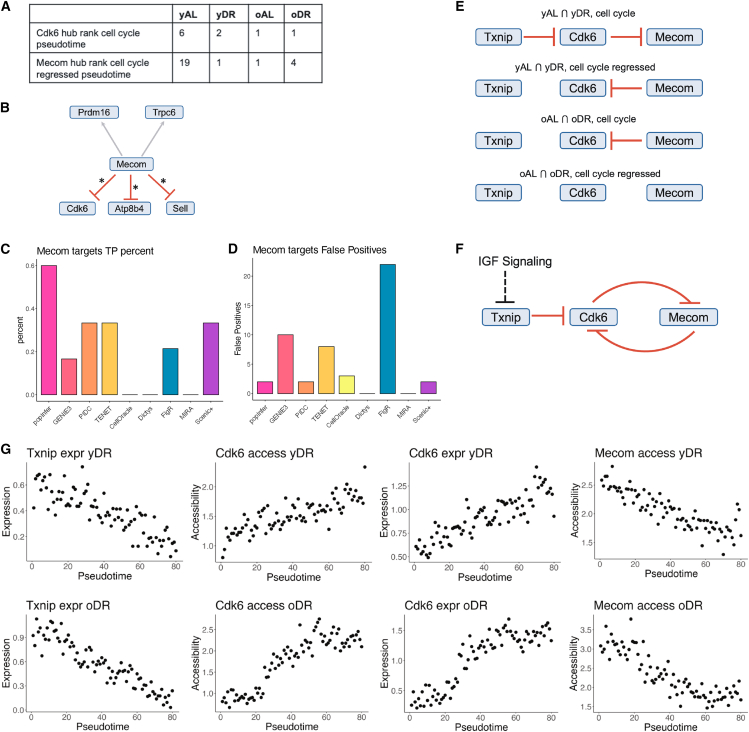
*popInfer identifies* the *regulation of HSC quiescence* via *IGF signaling* (A) Table of rankings of genes *Cdk6* and *Mecom* as network hubs, as inferred by popInfer. (B) Targets of *Mecom* predicted by popInfer for yAL, using pseudotime values computed from cell cycle regressed gene expression. Asterisk denotes genes that were differentially expressed in.^79^ (C) Percentage of inferred targets of *Mecom* in yAL that overlap with differentially expressed Mecom target genes from.^79^ (D) The number of false positives in the inferred targets of *Mecom* for each method. (E) Consensus networks of *Cdk6* and *Mecom* between different pairs of popInfer networks. (F) Consensus network between *Txnip*, *Cdk6*, and *Mecom*. Black dashed line denoting the effects of IGF signaling on the system is from the literature. (G) Plots of pseudocells over pseudotime (cell cycle not regressed), depicting the dynamics of *Txnip* expression, *Cdk6* accessibility, *Cdk6* expression, and *Mecom* accessibility for yDR and oDR.

In the networks predicted by popInfer, two genes stood out as hub regulators of the HSC-to-multipotent transition across all eight conditions—four experimental samples and two cell cycle conditions (Figure 4A). The first gene was the Mds1 and Evi1 complex locus (*Mecom*), a transcriptional regulator and oncogene. *Mecom* is a marker of HSCs in murine^71^ and human hematopoiesis.^67^^,^^72^^,^^73^ The second was cyclin-dependent kinase 6 (*Cdk6*), a protein kinase that acts primarily as a regulator of the cell cycle and associated HSC phenotypes.^74^^,^^75^ However, studies have implicated roles for *Cdk6* in the transcriptional regulation of various facets of HSC activity, including oncogenesis and quiescence^76^^,^^77^^,^^78^; functions that can act independently of its primary role as a kinase.^58^ For cell cycling pseudotime, popInfer identified *Cdk6* among the highest ranked network hubs across conditions. For cell cycle-regressed pseudotime, popInfer identified *Mecom* as a highly ranked hub gene in all samples except yAL (Figure 4A). popInfer also predicted direct interactions between *Mecom* and *Cdk6* (Figure 4B). Thus, later in discussion we analyze the networks involving these genes in greater depth.

As a means of assessment of networks inferred by popInfer, we compared predicted target genes of *Mecom* with differentially expressed genes (DEGs) that were observed upon EVI1 activation in hematopoietic progenitor cells.^79^ EVI1 is a transcription factor encoded by *Evi1*, one of the alternative transcripts of *Mecom*. We found that the majority (3/5) of the popInfer-predicted target genes of *Mecom* in yAL were differentially expressed in;^79^ moreover, popInfer correctly identified the sign of the interaction in all cases (Figure 4B). We compared the predictions made by popInfer with alternative GRN inference methods. Networks were constructed for RNA-only or multiomic GRN inference methods (see STAR Methods) and studied for their ability to predict target genes of *Mecom*. We retained 299 edges in each network as a direct comparison to the 299 edges detected in the popInfer network (with a gene interaction weight cutoff of 0.4). popInfer identified EVI1-associated DEGS^79^ with a success rate of almost twice the next best method (Figures 4C, S4, and 5), without sacrificing false positives (Figure 4D). This was true among both RNA-only methods and multiomic GRN inference methods, although in the case of the latter, only FigR and SCENIC+ identified any true Mecom targets, and FigR did so only with a high false positive rate (Figure 4D). We also assessed the impact of popInfer parameters on inferred networks. Alternative methods for pseudotime inference were overall concordant, although Slingshot found evidence of a branchpoint that is not supported by the data (Figure S6). Time and memory requirements were low for both genes and pseudocells until the number of genes grew large (Figure S7).

**Figure 5:**
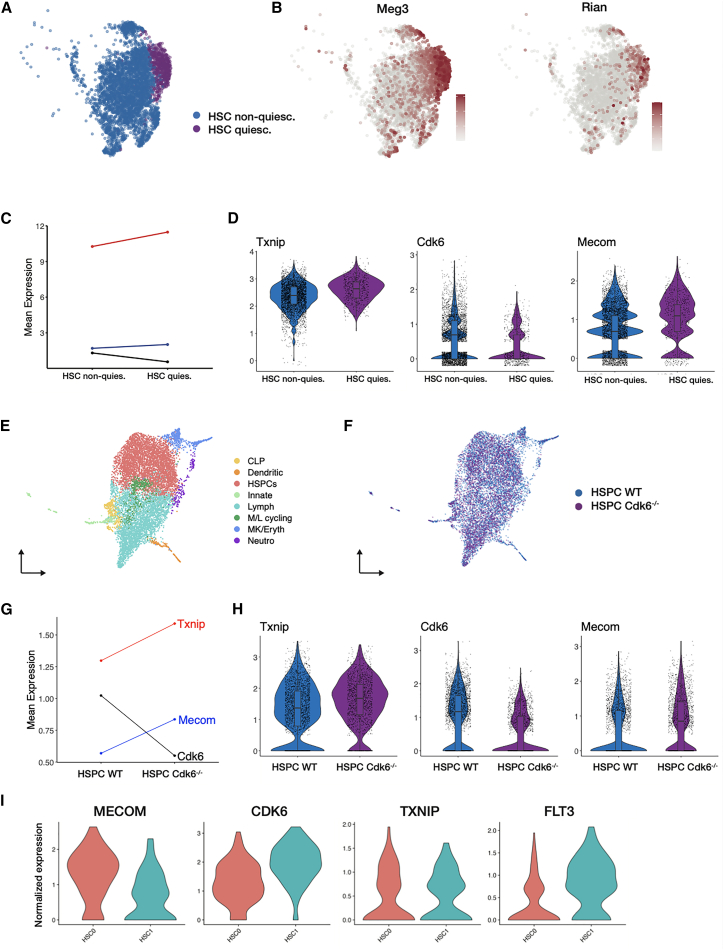
*Multiple independent datasets corroborate the popInfer-predicted dynamics* (A) UMAP of scRNA-seq data of myeloid-biased HSCs from Suo et al.^57^ Cells are colored by the groups compared in Suo et al., where cells in cluster 3 were identified as having high expression of quiescence markers. (B) UMAP of normalized expression of stem cell quiescence markers: *Meg3* and *Rian*. (C) Comparison of gene expression for *Txnip*, *Cdk6*, and *Mecom* between quiescent and non-quiescent HSC states. (D) Violin plots of the gene expression distributions between groups from (C). (E) UMAP of cell types identified by Mayer et al. in WT and CDK6^−/−^ HSCs.^58^ (F) Cells from both WT and KO contribute to HSPC clusters. (G) Comparison of gene expression for *Txnip*, *Cdk6*, and *Mecom* between WT and CDK6^−/−^ HSCs. (H) Violin plots of the gene expression distributions between groups from (C). (I) Comparison of gene expression across clusters HSC0 and HSC1 from Weng et al.^59^

Next, we identified consensus interactions between *Cdk6* and *Mecom* during the HSC-to-multipotent transition across multiple conditions. At a young age, for cell cycling pseudotime, the conserved network across dietary conditions consisted of *Cdk6* inhibition of *Mecom*, as well as Thioredoxin-interaction protein *Txnip* inhibition of *Cdk6* (Figure 4E). *Txnip* has been implicated in hematopoiesis in the context of regulating oxidative stress.^80^ For cell cycle-regressed pseudotime, the conserved network consisted of *Mecom* inhibition of *Cdk6* (Figure 4E). At old age, for cell cycling pseudotime, the conserved network consisted of *Mecom* inhibition of *Cdk6* (Figure 4E). For cell cycle-regressed pseudotime, there were no conserved interactions between control vs. dietary intervention in old age. The consensus network of interactions present in young hematopoiesis but which are lost with age is shown in Figure 4F (red edges). *Txnip* is negatively regulated by *Igf1*, of the insulin-like growth factor (IGF) pathway.^81^ IGF signaling plays an important role in regulating changes to HSC activity during aging.^82^^,^^83^^,^^84^ Previous literature supports specific interactions in the predicted consensus network, including the inhibition of *Cdk6* by *Txnip*, and the inhibition of *Cdk6* by *Mecom*.^79^^,^^85^

Deeper analysis of the gene expression and accessibility dynamics sheds light on how changes to the regulatory networks occur upon aging. The pseudocell-over-pseudotime dynamics show that in yDR there is a strong negative relationship between *Txnip* expression and *Cdk6* accessibility, as well as between *Cdk6* expression and *Mecom* accessibility (Figure 4G). In contrast, the relationships between these dynamic gene profiles in oDR samples are altered. While there is a similar pattern of *Txnip* expression over pseudotime, its correlation with *Cdk6* accessibility is much lower due to the *Cdk6* accessibility profile plateauing both at the start and at the end of pseudotime. Concordant with its accessibility, *Cdk6* expression also plateaus at the start and the end of pseudotime. In contrast, *Mecom* accessibility decreases close to linearly until late in pseudotime.

### Gene regulatory networks control of hematopoietic stem cell entry into/exit from quiescence is corroborated by multiple independent datasets

Above, we constructed a consensus GRN maintained across multiple conditions, whereby *Txnip* inhibits *Cdk6* and *Cdk6* and *Mecom* mutually inhibit one another during hematopoiesis in young mice (Figure 4F). This IGF-associated regulatory motif is lost with age. The age-associated loss of this mutual inhibitory motif that connects cell cycle regulation with HSC quiescence activity aligns with our understanding that HSC quiescence becomes harder to maintain as we age.^20^^,^^86^ Previously, we have shown that diet restriction-mediated suppression of IGF signaling can increase HSC quiescence.^16^ Our results here demonstrate the means by which this can occur: reduced IGF signaling upon DR leads to higher levels of *Txnip* and *Mecom*, reducing HSC cell cycle activity and driving stem cells toward a more quiescent state.

To assess the evidence for the gene regulatory network dynamics predicted by popInfer, we analyzed single-cell RNA-sequencing data of myeloid-biased HSCs from young (six-week-old) mice.^57^ In Suo et al. we clustered myeloid-biased HSCs and identified a subpopulation (cluster 3) that was enriched for IGF signaling markers. Here, we re-analyzed these data to compare cluster 3 with clusters 0, 4, 5, and 8 from Suo et al. (HSC subpopulations are not associated with upregulated IGF signaling). Relative to these clusters, we see that cluster 3 is enriched for stem cell quiescence markers *Meg3*^87^ and *Rian* (Figures 5A and 5B). According to their IGF signaling and quiescence states, cells in cluster 3 should be earlier along the HSC-to-multipotent differentiation trajectory than cells from the other HSC clusters. Thus, according to the popInfer-predicted network (Figure 4F), we would predict *Txnip* and *Mecom* to have higher expression in cluster 3, and *Cdk6* to have lower expression in cluster 3, relative to the other HSC clusters. These gene expression differences are observed exactly as predicted in the young myeloid-biased HSCs, whereby the shift from clusters 0,4,5,8 to cluster 3 is associated with increases in *Txnip* and *Mecom* and a decrease in *Cdk6* (Figures 5C and 5D). The differences in gene expression between HSC states were significant in all cases (two-sample Kolmogorov-Smirnov test: *p* = 4.17e-06 (*Txnip*); *p* = 2.2e-16 (*Cdk6*); *p* = 0.0016 (*Mecom*).

We also analyzed gene expression in the HSC quiescence network when it is perturbed by *Cdk6* knockout.^58^ Mayer et al. showed that *Cdk6* plays an important role in HSC self-renewal, consistent with the mutually inhibitory *Mecom—Cdk6* network predicted in this study. We re-analyzed the single-cell data reported in this study and clustered cells to identify stem cell and HSPC populations (Figure 5E), independent of genotype (Figure 5F). Within the HSPC population, we studied the expression of *Cdk6*, *Mecom*, and *Txnip* and found that the results recapitulated the profile above and matched the network results that popInfer predicts. In Cdk6^−/−^ HSPCs, as *Cdk6* expression decreases, we observed significant increases in the expression of *Mecom* and *Txnip* (Figures 5G and 5H).

Furthermore, we analyzed the expression of key genes in a dataset of human hematopoiesis^59^ according to the clustering provided in the original work, which found the two most abundant HSC states (“HSC0” and “HSC1”) to differ in their MECOM expression. The expression of CDK6 and TXNIP between these HSC states aligned with murine hematopoiesis and the patterns of expression predicted by popInfer (Figure 5I). We also see that FLT3 was significantly upregulated in HSC1 (the CDK6 high population), a highly relevant leukemic/stemness gene and a direct target of CDK6.^88^ The corroboration of multiomic data-based predictions with independent single-cell data supports our central hypothesis: that mutual inhibition between *Cdk6* and *Mecom* controls entry into/exit from HSC quiescence at a young age, facilitating the differentiation of HSCs into multipotent progenitors in the non-quiescent state. Previous evidence from the literature supports the role of *Cdk6* in ushering HSCs out of quiescence.^77^^,^^78^ Moreover, in human hematopoiesis where MECOM variants are associated with HSC-related disorders,^89^ perturbation of *MECOM* by CRISPR (to mimic a genetic disease caused by *MECOM* haploinsufficiency) found that *TXNIP*, the human ortholog of murine *Txnip*, is significantly downregulated with loss of *MECOM*,^67^ in agreement with the HSC quiescence network identified in this work.

Our results also resolve a contradiction that has been raised in the literature regarding the role of IGF signaling and aging. Young et al. demonstrated that an HSC aging phenotype could be observed in middle age (9–12 months old mice),^82^ and reported that the IGF1 stimulation of HSCs in middle age improved stem cell function. This stands seemingly in contrast to our previous work, which showed that a *reduction* in IGF1 signaling via DR improves stem cell function with age,^16^ which is in agreement with a growing body of literature indicating that caloric restriction in animals reduces senescence and improves longevity.^15^^,^^90^ Our data can resolve this paradox by considering the effects of IGF signaling in light of HSC quiescence. HSC stimulation via IGF1 allows stem cells to regain myeloid balance and repopulate the blood; however, we hypothesize that this functional recovery is short-term as stem cells must exit from quiescence for repopulation. Extended IGF1 stimulation would deplete the quiescent HSC pool that is required for healthy aging, thus leading to accelerated aging. Reduced IGF1 via DR at a young age thus impairs the short-term capacity of the HSC pool but improves it long-term by maintaining sufficient quiescent stem cells to confer anti-aging effects.

## Discussion

The ability to accurately predict GRNs that govern dynamic cell state transitions during cell differentiation and development could transform our understanding of cell fate decision-making with far-reaching consequences and applications. A persistent obstacle to achieving this goal has been the non-temporal nature of genomics data at single-cell resolution. Pseudotime has powerful applications, but it has been a wholly imperfect substitute for biological time.^48^ Joint multiomic data have revealed new gene regulatory mechanisms^91^^,^^92^ and here enable the inference of GRNs controlling dynamic transitions via the construction of pseudocells over pseudotime. Aided by the co,ntemporaneous measurements of regulator gene expression and target gene accessibility, popInfer predicts signed, directed gene regulatory interactions.

Through benchmarking on networks describing transitions during early hematopoiesis: HSCs to multipotent progenitors, or HSCs to granulocyte/monocyte progenitors, we showed that popInfer consistently outperforms alternative methods that rely on measurements of the gene expression alone. popInfer run on data excluding chromatin accessibility information (i.e., RNA to RNA prediction using pseudocells over pseudotime) attained similar performance to alternative RNA-only methods. Comparison of popInfer to alternative methods for GRN inference that use multiomic data found that popInfer identified sets of top regulators (transcription factors with the most targets) that were enriched for hematopoietic factors and more balanced (activating vs. inhibitory) than all other methods except for Dictys, which performed similarly. We note a fundamentally different assumption in popInfer compared with alternative multiomic GRN inference tools, which typically build base GRNs guided by the ATAC-seq^93^ and then separately infer network regulations based on gene expression. There is likely utility in the information gained from chromatin in these methods, yet they decouple the relationship between genome accessibility and target gene expression, which, as we have seen, hinders our ability to learn GRNs that capture interactions during dynamic cell state transitions. Quantifying the correspondence between the transcriptional dynamics that are captured by pseudotime and the chromatin dynamics^94^ ought to be explored in future work.

In application to early cell fate decisions during hematopoiesis, we studied hematopoietic stem and progenitor cells in young and old mice that were fed either *ad libitum* or a diet restricted. For each condition, we considered two approaches: either leaving in or regressing out the effects of the cell cycle. From these analyses, we discovered a core regulatory network predicted by popInfer, governed by the mutual inhibition of *Mecom* and *Cdk6*. This network regulated the transition of HSCs to multipotent cells by modulating quiescence. The network is also a target of IGF signaling, known to be reduced by diet restriction. Thus, we have demonstrated a mechanism by which HSC quiescence is increased upon diet restriction at young age,^16^ concordant with decreases in *Cdk6* and increases in *Mecom*. We tested the predicted GRN changes through analysis of scRNA-seq data of myeloid-biased HSCs from our previously published study.^57^ In comparing the expression patterns of HSCs enriched for quiescence markers vs. the non-quiescent HSC clusters, we recover exactly the relationships expected for our predicted GRN: with the higher expression of *Txnip* and *Mecom* and lower expression of *Cdk6* in quiescent HSCs. We were therefore able to corroborate our predictions from multiomic data using an independently collected dataset from a different sequencing modality (scRNA-seq).

In old age, we saw that the GRN motif (mutual inhibition of *Mecom* and *Cdk6* in response to IGF signaling) was lost. This aligns with the result that IGF1 levels decrease by middle age.^82^ As such, HSCs are less able to maintain quiescence. Moreover, the expression of *Igf2bp2*—an HSC-intrinsic activator of IGF signaling—is strongly diminished in aging HSCs.^57^ These HSC-extrinsic and intrinsic reductions in IGF signaling lead to the loss of IGF-dependent regulation of *Mecom* that we predict occurs in aged HSCs. Hematopoiesis during homeostasis is mostly driven by multipotent progenitors,^95^ emphasizing the importance of the quiescent HSC pool, from which cells exit rarely to divide and which is depleted with age.

In conclusion, popInfer for GRN inference uses multiomic data to overcome inherent challenges in dynamic inference from single-cell data, with wide applicability to the study of cell state transitions in development and regeneration. In application to multiomic data characterizing hematopoietic stem cells and progenitor cells, we have identified a regulatory network underlying the transition from HSCs to multipotent progenitor cells. The network consists of mutual inhibition between *Mecom* and *Cdk6*, influenced by the IGF signaling pathway via *Txnip*. Changes induced by diet restriction lead to an increase in HSC quiescence at a young age; however, this ability to increase HSC quiescence via *Mecom* upregulation with DR is lost with aging. This paves the way for future studies into mechanisms by which the loss of functional capacity of HSCs upon aging could be slowed or even reversed.

### Limitations of the study

We note some limitations of popInfer. The current model does not consider genome properties (e.g., TF motifs, binding sites, or genome shape^96^^,^^97^) in its analysis of gene accessibility, nor does it consider enhancer regions or other distal regulatory elements. Incorporation of additional genome features—such as peaks from enhancer regions—into a gene accessibility score could improve specificity, although the combinatorial complexity of such a model quickly becomes unwieldy. Another limitation of popInfer regards hyperparameter selection. While for the most part, popInfer-predicted networks were robust to the choice of the *α*, inferred networks may be sensitive to the number of pseudocells. There is an inevitable trade-off between noise (with many pseudocells) vs. over-smoothing and thus the loss of information (with few pseudocells). Choice of the number of pseudocells ought to be made with care and in light of the specific dynamics under investigation. In future work, the use of semi-supervised methods for pseudotime could help.^98^ If the pseudocell dynamics are discontinuous or ultrasensitive (which can result from, e.g., lineage branching in pseudotime), the assumptions upon which popInfer relies may no longer hold. popInfer sought to overcome inherent challenges with regression through the use of dynamic multiomic data for inference; however, it cannot rule out the possibility of inferring indirect as well as direct interactions. Here, model-based approaches to ameliorate statistical methods are a promising future direction.^34^^,^^99^ Central to popInfer is the assumption that a single network captures the cell fate decision that is characterized by the pseudotime dynamics. Alternative assumptions can be made: Dictys, for example, permits inference of both static and time-varying networks over pseudotime. In cases where the data comprise cell state transitions covering more ground (e.g., HSPC differentiation), it may become important to consider networks that change over time.

## Resource availability

### Lead contact

Further information and requests for resources should be directed to and will be fulfilled by the lead contact, Adam MacLean (email: macleana@usc.edu).

### Materials availability

This study did not generate new unique reagents.

### Data and code availability

• This article analyzes a new multiomic single-cell sequecing dataset that is available on the Gene Expression Omnibus (accession number: GSE229892).
• This article analyzes existing, publicly available data. These accession numbers for the datasets are listed in the key resources table.
• Implementation of popInfer in R is available at https://github.com/maclean-lab/popInfer under an MIT license and archived on Zenodo at: https://zenodo.org/records/16754699.
• Any additional information required to reanalyze the data reported in this article is available from the lead contact.

## Acknowledgments

The authors would like to thank Ivonne Görlich and Marco Groth (FLI sequencing facility) for help with the multiome assay. ALM acknowledgments support from the 10.13039/100000002National Institutes of Health (R35GM143019) and the 10.13039/100000001National Science Foundation (DMS2045327 and MCB2421941).

## Author contributions

M.K.R.: conceptualization, investigation, analysis, methodology, and software. M. Behrends: investigation, analysis, and methodology. YC: investigation, analysis, and methodology. J.M.: investigation, analysis, and software. N.K.: investigation, analysis, and software. N.G.: investigation and analysis. D.G.: investigation and analysis. M. Bens: investigation. L.X.: methodology and software. Z.J.: investigation and analysis. K.L.R.: conceptualization, analysis, methodology, and supervision. A.L.M.: conceptualization, analysis, methodology, software, and supervision. M.K.R. and A.L.M. wrote the article with input from all authors.

## Declaration of interests

The authors declare no competing interests.

## STAR★Methods

### Key resources table

**Table:** 

REAGENT or RESOURCE	SOURCE	IDENTIFIER
**Antibodies**		

Biotin TER 119	BioLegend	Cat#116204; RRID: AB_313705
Biotin GR1	BioLegend	Cat#108404; RRID: AB_313369
Biotin CD11b	BioLegend	Cat#101204; RRID: AB_312787
Biotin B220	BioLegend	Cat#103204; RRID: AB_312989
Biotin CD4	BioLegend	Cat#100508; RRID: AB_312711
Biotin CD8a	BioLegend	Cat#100704; RRID: AB_312743
CD150 Brilliantviolet 605	BioLegend	Cat#115927; RRID: AB_11204248
c-kit APC	BioLegend	Cat#105812; RRID: AB_313221
SCA1 (Ly-6A/E) PE	BioLegend	Cat#108108; RRID: AB_313345
Streptavidin APC-Cy7	BioLegend	Cat#405208
CD34 FITC	Invitrogen	Cat#11-0341-85; RRID: AB_465022
CD16/32 PE-Cy7	BioLegend	Cat#101318; RRID: AB_2104156

**Biological samples**		

Bone marrow obtained from mice hind limbs, fore limbs, and spines	This study	N/A

**Chemicals, peptides, and recombinant proteins**		

20x Nuclei Buffer	10x Genomics	PN-1000285
RNase inhibitor	Promega	N2511/N251A
Nuclease-free water	Promega	P119A
BSA	PanReac AppliChem	A1391,0025
Tris-HCl pH 7.4	Roth	4855.7
NaCl	Roth	3957.5
MgCl2	Merck	814733
Nonidet P40 Substitute	Roche	11332473001
Digitonin	Promega	G9441
Tween 20	Roche	11332465001

**Critical commercial assays**		

Agilent High Sensitivity DNA Kit	Agilent	Cat#5067-4626
D5000 ScreenTape Assay	Agilent	Cat#5067–5588, Cat#5067–5589, Cat#5067-5590
DNA 7500 Assay	Agilent	Cat#5067-1506
Chromium Next GEM Single Cell Multiome ATAC + Gene Expression Reagent Bundle, 16 rxns	10x Genomics	PN-1000283
Chromium Next GEM Chip J Single Cell Kit	10x Genomics	PN-1000230
Single Index Kit N Set A	10x Genomics	PN-1000212
Dual Index Kit TT Set A	10x Genomics	PN-1000215
NovaSeq 6000 SP Reagent Kit	Illumina	Cat#20028401
NovaSeq 6000 S1 Reagent Kit	Illumina	Cat#20028319

**Deposited data**		

Raw and processed single-nuclei multiomic data	This paper	GEO: GSE229892
Mouse reference genome NCBI build 38, GRCm38	Genome Reference Consortium	https://www.ncbi.nlm.nih.gov/grc/mouse
Mouse scRNA-seq gimyeloid-biased HSC dataset	Suo et al.^57^; https://doi.org/10.1182/blood.2021012197	GEO: GSE166176
Mouse scRNA-seq WT and CDK6−/− HSCs dataset	Mayer et al.^58^; https://doi.org/10.1182/blood.2023021985	ArrayExpress: E-MTAB-13149
Human scRNA-seq HSC dataset	Weng et al.^59^; https://doi.org/10.1038/s41586-024-07066-z	GEO: GSE219015

**Experimental models: Organisms/strains**		

Mouse: C57BL/6J wildtype: Black mouse, a (a/a) non agouti - MHC: Haplotype H2^b^	The Jackson Laboratory	Cat#000664

**Software and algorithms**		

FACSDiva	BD Biosciences	https://www.bdbiosciences.com/
FlowJo	BD Biosciences	https://www.bdbiosciences.com/en-us
R 4.2.1	The R Foundation	https://www.r-project.org
Python 3.11	Python Software Foundation	https://www.python.org
10X Genomics Cell Ranger ARC v2.0.0	10X Genomics	https://www.10xgenomics.com/support/software/cell-ranger-arc/latest
Seurat v3.2.3	Hao et al.^54^; https://doi.org/10.1016/j.cell.2021.04.048	https://github.com/satijalab/seurat
ArchR v1.0.2	Granja et al.^100^; https://doi.org/10.1038/s41588-021-00790-6	https://github.com/GreenleafLab/ArchR_2020
SoupX v1.3.6	Young et al.^101^; https://doi.org/10.1093/gigascience/giaa151	https://github.com/constantAmateur/SoupX
DoubletFinder	McGinnis et al.^102^; https://doi.org/10.1016/j.cels.2019.03.003	https://github.com/chris-mcginnis-ucsf/DoubletFinder
popInfer v0.1	This paper	https://github.com/maclean-lab/popInfer; https://doi.org/10.5281/zenodo.16754699
BEELINE v1.0	Pratapa et al.^43^; https://doi.org/10.1038/s41592-019-0690-6	https://github.com/murali-group/BEELINE
GENIE3 v1.31.0	Huynh-Thu et al.^25^; https://doi.org/10.1371/journal.pone.0012776	https://github.com/vahuynh/GENIE3
PIDC v0.1.1	Chan et al.^31^; https://doi.org/10.1016/j.cels.2017.08.014	https://github.com/Tchanders/network_inference_tutorials
TENET v2.4	Kim et al.^30^; https://doi.org/10.1093/nar/gkaa1014	https://github.com/neocaleb/TENET
SINCERITIES v2.0	Papili Gao et al.^61^; https://doi.org/10.1093/bioinformatics/btx575	https://github.com/CABSEL/SINCERITIES
CellOracle v0.10.12	Kamimoto et al.^36^; https://doi.org/10.1038/s41586-022-05688-9	https://github.com/morris-lab/CellOracle
Dictys v1.1.0	Wang et al.^41^; https://doi.org/10.1038/s41592-023-01971-3	https://github.com/pinellolab/dictys
SCENIC+ v1.0a2	Bravo González-Blas et al.^40^; https://doi.org/10.1038/s41592-023-01938-4	https://github.com/aertslab/scenicplus
FigR v0.1.0	Kartha et al.^65^; https://doi.org/10.1016/j.xgen.2022.100166	https://buenrostrolab.github.io/FigR/
MIRA v2.1	Lynch et al.^66^; https://doi.org/10.1038/s41592-022-01595-z	https://github.com/cistrome/MIRA

### Experimental model details

#### C57BL/6J wildtype mice

C57BL/6J wildtype black mice, a (a/a) non agouti - MHC: Haplotype H2^*b*^, were obtained from the Jackson Laboratory (Cat#000664). All mouse experiments were approved by the state Government of Thuringia under the application “FLI19-009”. Male and female C57BL/6J mice were obtained from Janvier, bred in the FLI’s animal facility and kept in groups of 3–5 same sex littermates on a 12:12 h light:dark cycle in 20°C–24°C and 40–60% air humidity. The animal facilities were specific pathogen free and the mice’s cages (Tecniplast) were either individually ventilated or provided with a filter top.

### Method details

#### Joint multiomics experimental methods

##### Animal experiments on dietary interventions

Young (∼6 months old) and aged (∼24 months old) female C57BL/6J mice were randomly distributed into age- and weight-matched groups. The mice were single-housed and fed with chow prepared from commercially available powder (VRF1, SNIFF). Over the course of the first week the food intake of the individual mice was measured. At the beginning of the second week the dietary restriction (DR) group were fed once a day shortly before the onset of darkness with a food portion corresponding to 70% of the normal intake of the respective mouse. Animals were scored and weighed every other day.

##### Cell isolation and staining

The mice’s hind limbs (including hip bones joints), forelimbs and spines were dissected, cleaned, and crushed in 2% FBS using mortar and pestle. Bone marrow cells were incubated with APC-conjugated anti-c-Kit antibody, and c-Kit+ cells were enriched using anti-APC magnetic beads (MACS Milteny Biotec 130-09-855) and LS columns. C-kit positive cells were then stained with an antibody mix against mature cells (Table S1) for 30 min and overnight with a second fluorescent AB mix to stain markers used to discern different populations of HSPC (Table S2). The cells were sorted on an ARIA III cell sorter (BD bioscience) according to the markers in Table S3. 50,000 LSK cells per mouse were sorted and cells from 2 mice per group were pooled.

##### Nuclei isolation

Cells were collected by centrifugation (10′, 300g, 4°C), the supernatant was removed and the cells were resuspended in 50 *μL* 0.04% sterile filtered BSA in 1x PBS. Cells were pelleted (10′, 300g, 4°C), supernatant was discarded and nuclei isolation was conducted in accordance with the manufacturer’s protocol. In brief, 45 *μL* ice-cold lysis buffer (Table S4) were added to the cells. Cells were pipetted up and down 3 times and incubated on ice for 3’. Subsequently 50 *μL* ice-cold wash buffer (Table S5) were added. The samples were centrifuged for 5′ at 500g and 4°C and 95 *μL* supernatant were removed. The nuclei were washed with 45 *μL* ice-cold 1x nuclei buffer (Table S6) and centrifuged for 5′ at 500g and 4°C. 40 *μL* supernatant were removed with a 100 *μL* pipette, the remainder with a 10 *μL* pipette. The nuclei pellet was resuspended in 7 *μL* ice-cold 1x nuclei buffer.

##### 10x multiome protocol

1 *μL* aliquot of the nuclei suspension were stained with DAPI and analyzed with flow cytometry (LSRFortessa BD Bioscience) to measure final nuclei stock concentration was. Nuclei stock concentrations ranged from 3178 to 8887 nuclei/*μl*. Samples were immediately processed for scATAC-seq and scRNA-seq targeting 10,000 nuclei per sample. Samples were loaded separately onto the channels of the 10x Genomics Chromium Controller and processed with Single Cell Multiome ATAC + Gene Expression (v1 chemistry) following the standard manufacturer’s protocol (Document Number CG000338 Rev E). For ATAC-seq libraries, 7 cycles were used for the Sample Index PCR reaction and final libraries were evaluated using D5000 ScreenTape (Agilent 4200 TapeStation System) and DNA 7500 (Agilent 2100 Bioanalyzer). For scRNA-seq libraries, cDNA was amplified by 7 cycles and the total yield of cDNA was assessed on High Sensitivity DNA Assay (Agilent 2100 Bioanalyzer) resulting on average in 168 ng. A total of 13 cycles was then used for the Sample Index PCR reaction and final libraries were evaluated using D5000 ScreenTape (Agilent 4200 TapeStation System). Each type of library was pooled and sequenced using Illumina NovaSeq6000 System on SP flowcells.^103^ scATAC-seq: Read 1 and Read 2 53 bp for DNA Insert, i7 8 bp for sample index and i5 24 bp for 10x Barcode and Spacer. scRNA-seq: Read 1 28 bp for 10x Barcode and UMI; Read 2 90 bp for Insert, i7 and i5 10 bp for sample index. The initial analysis with 10x Genomics Cell Ranger 2.0.0 (bcl2fastq v2.20.0.422) and corresponding pre-built mouse reference package (mm10-2020-A) estimated 2,576 to 5,833 nuclei per sample with at least 50,000 reads per nuclei.

#### Joint multiomics data analysis pipeline

##### Data preprocessing and quality control

yAL, yDR, oAL, and oDR single-nucleus multiome ATAC + RNA data were processed using 10X Genomics Cell Ranger ARC (v2.0.0) mapped to the GRCm38 reference genome. Seurat (v3.2.3) and ArchR (v1.0.2) packages were used for all further analysis.^54^^,^^100^

For oAL, we began with 4754 cells. We first removed ambient RNA using SoupX,^101^ manually setting the contamination to 10%. Using ArchR for ATAC quality control, cells with TSS enrichment <5.0 or number of fragments <1200 were removed. Using Seurat for RNA quality control, we remove cells with transcript counts >15000 or <1500, cells with number of features <1300, or cells with mitochondrial percentage >20%. After this initial quality control, 4082 cells remained. Lastly, we ran Doublet Finder^102^ with 3% doublet formation rate and PC neighborhood size *pK* = 0.29, removing 122 doublets and leaving us with a final set of 3960 cells (Figure 1A).

For oDR, we began with 2576 cells. We first removed ambient RNA using SoupX, manually setting the contamination to 10%. Using ArchR for ATAC quality control, cells with TSS enrichment <8.0 or number of fragments <2000 were removed. Using Seurat for RNA quality control, we remove cells with transcript counts >10000 or <1000, cells with number of features <500, or cells with mitochondrial percentage >35%. After this initial quality control, 2299 cells remained. Lastly, we ran Doublet Finder with 3% doublet formation rate and PC neighborhood size *pK* = 0.17, removing 69 doublets and leaving us with a final set of 2230 cells.

For yAL, we began with 4185 cells. We first removed ambient RNA using SoupX, manually setting the contamination to 10%. Using ArchR for ATAC quality control, cells with TSS enrichment <5.0 or number of fragments <2000 were removed. Using Seurat for RNA quality control, we remove cells with transcript counts >9000 or <1000, cells with number of features <700, or cells with mitochondrial percentage >25%. After this initial quality control, 3423 cells remained. Lastly, we ran Doublet Finder with 3% doublet formation rate and PC neighborhood size *pK* = 0.29, removing 103 doublets and leaving us with a final set of 3320 cells.

For yDR, we began with 3616 cells. We first removed ambient RNA using SoupX, manually setting the contamination to 10%. Using ArchR for ATAC quality control, cells with TSS enrichment <5.0 or number of fragments <3000 were removed. Using Seurat for RNA quality control, we remove cells with transcript counts >9000 or <700, cells with number of features <500, or cells with mitochondrial percentage >40%. After this initial quality control, 3146 cells remained. Lastly, we ran Doublet Finder with 3% doublet formation rate and PC neighborhood size *pK* = 0.27, removing 94 doublets and leaving us with a final set of 3052 cells.

##### Hematopoietic cell subpopulation clustering and peak calling

For all datasets, transcript counts were transformed by regressing out *G*2*M* and *S* phase cell cycle scores (from the CellCycleScoring Seurat function) using the Seurat function SCTransform prior to clustering (Figures 1B and 1D). In all datasets, the first 30 principal components were used for louvain clustering and Uniform Manifold Approximation and Projection (UMAP) in Seurat. Clustering resolution was set to 0.25 for *oAL* and *oDR*, 0.35 for *yAL*, and 0.31 for *yDR*, resulting in 6 distinct clusters in oAL, 7 distinct clusters in oDR and yAL, and 5 clusters in yDR. Corresponding cell types were annotated using canonical hematopoietic cell type markers. For peak calling, all four datasets were used to create a single ArchR object. Using the clusters defined using the RNA, the two smallest clusters in both *oAL* and *oDR* were removed prior to peak calling and the smallest cluster in *yDR* was removed prior to peak calling. Pseudo-bulk replicates were made in ArchR using the addGroupCoverages function with maxReplicates set to 10 and groupBy set to the samples and clusters defined using the RNA data. The pseudo-bulk replicates were used for peak calling via the addReproduciblePeakSet ArchR function.

#### popInfer model for gene regulatory network inference

##### Feature selection for network inference

To select features to use as input to the gene regulatory network inference model, we first identified genes that were differentially expressed between the HSC and multipotent progenitor clusters for each of the four samples using Seurat’s FindMarkers function. We defined a gene to be differentially expressed if it’s adjusted *p*-value was less than 0.05. We then took the union over these four sets of DEGs, giving us 107 genes. From this set of genes, we removed three genes that weren’t found in the reference chromatin annotation. The remaining 104 genes were used as the input features to the network inference method. For the transition from HSCs to GMPs in oAL, we used the 565 differentially expressed genes between the HSC and GMP clusters as the input features.

##### Pseudotime ordering and pseudocell construction

On each dataset, DPT^70^ was applied to the RNA assay to assign pseudotime values. We tested the concordance between DPT and alternative choices of pseudotime inference algorithm (Slingshot or Monocle^104^^,^^105^) and found overall that high correlation between methods, although slingshot found evidence of a branchpoint during the HSC to MPP transition that we do not think is supported by the data (Figure S6). The root cell of DPT was set to be the cell with the highest expression of the sum of Mecom and Mpl, two canonical HSC markers. For yAL, the root cell was defined as the cell with the fourth highest expression of the sum of Mecom and Mpl because the top three ranking cells either did not lie in the HSC cluster or were on the border between the HSC cluster and another cluster in the UMAP. The expression of the top 2000 variable features was used as input to DPT.

For each dataset, two different pseudotime assignments were produced: one that was computed on expression counts that included cell cycle effects and one that was computed on cell cycle regressed expression counts (Figures 1C and 1E). DPT^70^ was therefore applied twice to each sample. The first iteration of DPT was run on counts that were transformed after quality control using the Seurat function SCTransform. The second iteration of DPT was run on counts that were transformed by regressing out *G*2*M* and *S* phase cell cycle scores (from the CellCycleScoring Seurat function) using the Seurat function SCTransform.

For each dataset and for each pseudotime assignment, we constructed pseudocells, conceptually similar to other “metacell” approaches^106^^,^^107^ but distinctly defined. Suppose there are *N* total cells ordered by pseudotime which are to be paritioned into *n* similarly sized pseudocell bins. In order to use all of the available *N* cells, we define *n* bins with potentially uneven numbers of cells that differ by at most by one additional cell. We define the first *N* mod *n* bins contain $\left\langle \frac{N}{n}\right\rangle +1$ cells and the remaining *N*-(*N* mod *n*) bins contain $\left\langle \frac{N}{n}\right\rangle$ cells. Throughout this work, we use *n* = 80 as this number of pseudocell bins seemed to be able to summarize the dynamics of the trajectory, removing some noise/variability while not over-averaging. Selecting *n* values that were too small/large resulted in drops in performance. In general, we have found that selecting *n* that yields around 30–40 cells per pseudocell is optimal. The time and space requirements for running popInfer depend primarily on the number of genes selected for inference as opposed to the number of pseudocells (Figure S7).

Pseudocell expression (*x*^*p*^) was defined as the average of the expression of the *k* cells within it’s corresponding bin:

$$x^{p}=\frac{1}{k}\sum_{j=1}^{k}x_{j}^{s}$$

where $x_{1}^{s},x_{2}^{s},\ldots x_{k}^{s}$ are the expression values of the cells in the pseudocell bin.

For constructing pseudocell gene accessiblity scores, we used ArchR’s GeneScoreMatrix function. Using the default parameters, gene scores are computed using genes in the gene body and 5 kb upstream of the TSS. Pseudocell accessibility (*y*^*p*^) was defined as the average of the expression of the *k* cells within it’s corresponding bin:

$$y^{p}=\frac{1}{k}\sum_{j=1}^{k}y_{j}^{s}$$

where $y_{1}^{s},y_{2}^{s},\ldots y_{k}^{s}$ are the ArchR GeneScoreMatrix accessibility scores of the cells in the pseudocell bin for pseudocell *p*.

##### Pseudotime-ordered pseudocell (POP) inference model

From our pre-processing, we have *n* pseudocells, for which we have pseudocell expression scores $x_{1}^{p},x_{2}^{p},\ldots ,x_{n}^{p}$ and pseudocell gene accessibility scores $y_{1}^{p},y_{2}^{p},\ldots ,y_{n}^{p}$. From our feature selection, we have genes *G*. For each gene *g*∈*G*, we run a lagged LASSO regularized linear regression model using glmnet^108^:

$$\min_{\beta_{0},\beta }\frac{1}{n}\sum_{i=1}^{n}{\left(y_{i}^{p}\left(g\right)-\beta_{0}-{\left(x_{i}^{p}\right)}^{T}\beta \right)}^{2}+\lambda_{g}{\left\|\beta \right\|}_{1}$$

where *λ*_*g*_ is selected via the optimization:

$$\lambda_{g}=\min_{\lambda }\left|\alpha \frac{MSE_{\lambda }}{\left(MSE\;\text{trivial}\right)}-\left(1-\alpha \right)\frac{{\left(\#\;\text{nonzero}\;\beta \right)}_{\lambda }}{{\left(\#\;\text{nonzero}\;\beta \right)}_{\lambda_{MSE}}}\right|$$

where *α*∈[0,1], *MSE*_*λ*_ is the MSE of the LASSO model for *λ*, *MSE* trivial is the MSE when we have a trivial model (*β* = 0), (# nonzero β)_λ_ is the number of nonzero coefficients of the LASSO model for a given *λ* value, and *λ*^*MSE*^ is the value of *λ* for which the LASSO model achieves optimal MSE. We implement gene-specific sparsity parameters (*λ*_*g*_) because we found that using the same *λ* value across all genes resulted in inconsistent levels of sparsity (relative to the sparsity in the optimal MSE model for that gene). The optimization we defined thus allows for a better control over global sparsity of the model. We evaluate this optimization for a given *α* value by running glmnet for 200 *λ* values and solving.^108^

After running for a fixed *α* value, we define a *G*×*G* output matrix,

$$W_{\alpha }\left(g_{i},g_{j}\right)=\left\{ \begin{array}{cc} 1, & \;\text{if}\;\beta_{g_{i},g_{j}}>0\\ -1, & \;\text{if}\;\beta_{g_{i},g_{j}}<0\\ 0, & \;\text{otherwise}\; \end{array}\right.$$

for all *g*_*i*_, *g*_*j*_∈*G*, *i*≠*j*. Finally, we run for a sequence of *α* values *α*_1_,*α*_2_, …,*α*_*s*_∈[0,1] and define the final output of popInfer to be the weight matrix:

$$W=\frac{1}{s}\sum_{j=1}^{s}W_{\alpha j}\text{.}$$

In this work, we use the sequence of *α* values: *α*∈{0.001,0.002, …,0.4} with the exception of the oAL GMP branch testing, for which we used the sequence of *α* values: *α*∈{0.001,0.002, …,0.6}. We select *α* values closer to 0 because we expect GRNs to be sparse and according to our defined optimization problem, values of *α* closer to zero will be more sparse. Averaging over the results of a sequence of *α* values as opposed to only running for a single value of *α* is helpful in a number of situations, such as when a gene has a nonzero value of *β* only a few times by chance or if a gene interaction has inconsistent predicted sign (activating/inhibiting) for different *α* values. In both of these examples, sporadic or inconsistent relationships between a predictor and target gene will be mitigated by running for a sequence of *α* values.

#### GRN benchmarking

##### RNA-based methods

GENIE3, TENET, and SINCERITIES were run using the gene expression counts from the multiome data. The DPT pseudotime values were also used as input to TENET. For SINCERITIES, cells were grouped into 10 bins along pseudotime. These methods were run according to the directions in their documentation, and thus were not applied to pseudocells as in popInfer. However, a comparison of applying such methods to both single-cell and pseudobulked data would be an interesting exploration.

##### CellOracle

CellOracle^36^ was run from the author’s provided Docker image. A base GRN was first constructed using the R package Cicero^93^ to process scATAC-seq data, which was used to identify co-accessible chromatin regions. A CellDataSet object was constructed using the output from the CellRanger output. With the exception of filtering cells by peak count, the following steps were run as recommended: remove zero-read cells, LSI, and dimension reduction with UMAP. Cicero was run using the final object and the provided coordinate file for the mm10 reference genome to generate a co-accessibility matrix linking chromatin peaks. Co-accessible peaks were annotated and filtered using the CellOracle workflow, with transcription start site (TSS) information built into a CellOracle motif analysis module for the mm10 genome. TSS info was combined with Cicero connections, and peaks with weak co-accessibility scores were filtered out. The complete base GRN was exported following default motif calling in mm10 via gimmemotifs. An AnnData object containing pre-filtered cells and genes as described above (2422 cells and 104 genes in yAL) was used as input for scRNA-seq modeling. Cell-types (HSC or MPP) were assigned based on prior clustering information. The AnnData object was normalized and log-transformed. GRN inference was then performed in CellOracle with the constructed AnnData object and the base GRN. Edges with a *p*-value <0.05 were kept.

##### Dictys

Dictys^41^ was run using a Conda environment created based on the author’s instructions. Input data was prepared using the provided Dictys helper script expression_mtx.py on the CellRanger output. The bam file containing all ATAC-seq reads was split into per-cell bam files using the helper script split_bam.sh. Clustering and cell-annotation information from the popInfer analysis were then applied in Scanpy and subsequently used to create a cluster file in the required format (clusters.csv). Motifs for mm10 in the HOMER format was obtained from HOCOMOCO. The mm10 reference genome was extracted using the provided homer helper script. The bed file of gene regions and strand information to locate transcription start sites for the mm10 genome was obtained from Ensembl. Requisite makefiles were prepared using standard steps outlined in the Dictys documentation. No parameters were adjusted with the exception of setting the genome to mm. The network inference step was run on the static setting, to output the resulting h5 file that contains TF regulatory activity.

##### SCENIC+

SCENIC+^40^ was run using the authors’ pre-built conda environment. Pseudobulk ATAC-seq profiles were generated from the 10x fragments file. This file was filtered to only include barcodes matching the cell subset used in the popInfer analysis. Cell types were annotated based on prior clustering information. The pseudobulk profiles were used to call individual peaks for each cell type, these were then merged into a consensus peak set, which represents the union of accessible regions across all cell types of interest. This set of consensus regions is used downstream for topic modeling and differential accessibility analysis. A cisTopic object was created using the 10x CellRanger output and the blacklist file for the mm10 genome provided by SCENIC+. This was used as input for topic modeling. Candidate enhancer regions were inferred via binarization of region-topic probabilities and calculation of differentially accessible regions by cell type. Motif enrichment analysis was done using pyCistarget. The files for the mm10 motif rankings, scores, and annotations used by pyCistarget were used as provided in the documentation of SCENIC+ with default parameters. The Cistopic object and motif information were combined with the AnnData object as defined above to instantiate an SCENIC+ object. The SCENIC+ GRN inference step was run using a list of murine transcription factors (file provided by the authors). Parameters were set to defaults and the resulting eRegulons were filtered according to the recommended filtering steps provided in the documentation.

##### FigR

FigR^65^ was run in R with default parameters using a SummarizedExperiment object containing the gene expression counts from the initial Seurat object and the peak matrix from the ArchR object. Peak-gene correlations were filtered by pvalZ <0.05. Domain of open chromatin genes were defined as having at least 4 significant gene-peak correlations.

##### MIRA

MIRA is a suite of tools for regulatory analysis on multiomics data via topic modeling.^66^ MIRA provides means to infer GRNs via regulatory potential modeling to map TF-target gene relationships from gene topics. MIRA was run using a Conda environment created based on the author’s listed dependencies. A scanpy object of the multiome data was created directly from the 10X filtered feature matrix.The object was then subsetted to the same set of cells as in the original analysis. Per the recommendations in the documentation, the number of peaks used for the encoder network was down-sampled to reduce memory usage. Via evaluation of the topic contributions, the number of topics to model was set to 6. The model was further refined the Bayesian optimization strategy.

### Quantification and statistical analysis

#### Network assessment using BEELINE

Networks inferred by various tools were compared using the ChIP-atlas benchmark made available in BEELINE.^43^ Network quality was assessed via the Area Under the Early Precision Recall Curve (AUEPRC), which is the area under the precision recall curve for a restricted range of recall values, normalized by the total recall range. We focused on this restricted range since we expect true networks to be sparse relative to the number of possible interactions. Under these conditions, AUEPRC is well-suited to assess performance of each method, specifically, to assess whether methods perform well on their highest-ranked network interactions. For the HSC to multipotent transition in yAL, we evaluate AUEPRC over recall values in the range [0, 0.1].

#### Gene expression analyses

Differential gene expression between cell types was assessed via Wilcoxon rank sum or Kolmorogov-Smirnov tests, at a significance level of 0.05 with a Bonferroni multiple testing correction applied.
