# Supplementary material for: Gene regulatory network inference with popInfer reveals the dynamic regulation of hematopoietic stem cell quiescence
Source: iScience. 2025 Nov 11;28(12):114010. doi: 10.1016/j.isci.2025.114010 (PMC12757588; doi:10.1016/j.isci.2025.114010)
Supplement: Document S1. Figures S1–S5 and Tables S1–S6 [file mmc1.pdf]

iScience, Volume *28*

Supplemental information

Gene regulatory network inference with popInfer reveals the dynamic regulation of hematopoietic stem cell quiescence

Megan K. Rommelfanger, Marthe Behrends, Yulin Chen, Jonathan Martinez, Nikith Kurella, Nino Geisler, Deepthi Guturu, Martin Bens, Lingyun Xiong, Zijin Xiang, K. Lenhard Rudolph, and Adam L. MacLean

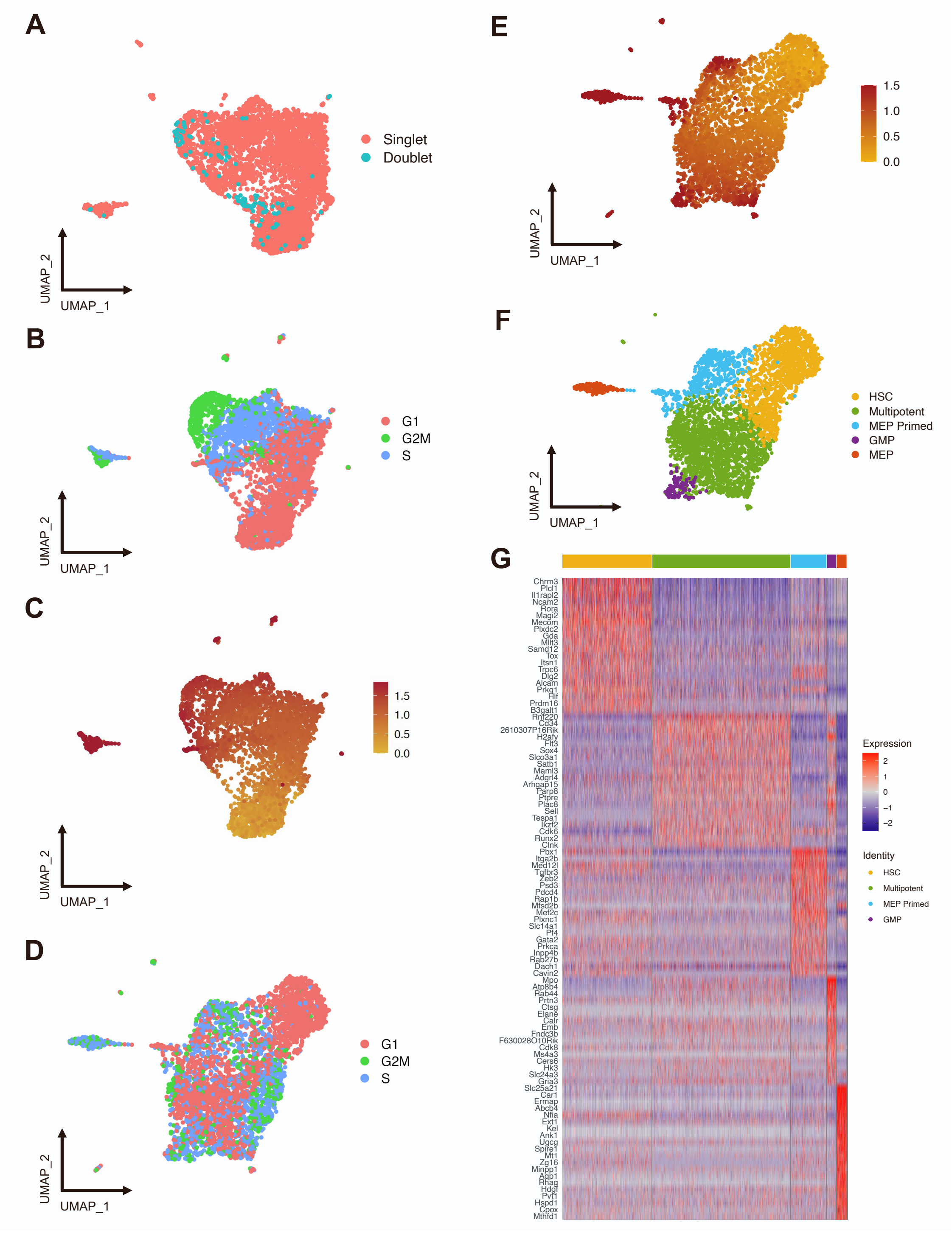
A
Singlet
Doublet
UMAP_2
UMAP_1
B
G1
G2M
S
UMAP_2
UMAP_1
C
1.5
1.0
0.5
0.0
UMAP_2
UMAP_1
D
G1
G2M
S
UMAP_2
UMAP_1
E
1.5
1.0
0.5
0.0
UMAP_2
UMAP_1
F
HSC
Multipotent
MEP Primed
GMP
MEP
UMAP_2
UMAP_1
G
Chrm3
Plcl1
Il1rapl2
Ncam2
Rora
Magi2
Mecom
Plxdc2
Gda
Mllt3
Samd12
Tox
Itsn1
Trpc6
Dlg2
Alcam
Prkg1
Hlf
Prdm16
B3galt1
Rnf220
Cd34
2610307P16Rik
H2afy
Flt3
Sox4
Slco3a1
Satb1
Maml3
Adgrl4
Arhgap15
Parp8
Ptpre
Plac8
Sell
Tespa1
Ikzf2
Cdk6
Runx2
Clnk
Pbx1
Itga2b
Med12l
Tgfbr3
Zeb2
Psd3
Pdcd4
Rap1b
Mfsd2b
Mef2c
Plxnc1
Slc14a1
Pf4
Gata2
Prkca
Inpp4b
Rab27b
Dach1
Cavin2
Mpo
Atp8b4
Rab44
Prtn3
Ctsg
Elane
Calr
Emb
Fndc3b
F630028O10Rik
Cdk8
Ms4a3
Cers6
Hk3
Slc24a3
Gria3
Slc25a21
Car1
Ermap
Abcb4
Nfia
Ext1
Kel
Ank1
Ugcg
Spire1
Mt1
Zg16
Minpp1
Aqp1
Rhag
Hdgf
Pvt1
Hspd1
Cpox
Mthfd1
Expression
2
1
0
−1
−2
Identity
HSC
Multipotent
MEP Primed
GMP

Figure S1: Overview of single-nucleus multiome data preprocessing workflow in oAL. (A) UMAP of DoubletFinder identified singlets and doublets in oAL. (B) After removal of doublets, UMAP of oAL data where cells are colored by cell cycle phase. Cell cycle effects appear significant, leading to cells clustering by phase. (C) UMAP of oAL data colored by pseudotime value. Pseudotime values greater than 2.0 were set to be 2.0 in order to clearly see the color gradient on the plot. (D) UMAP of oAL data after cell cycle effects are regressed. (E) UMAP of cell cycle regressed oAL data colored by pseudotime value. Pseudotime values greater than 1.5 were set to be 1.5 in order to clearly see the color gradient on the plot. (F) Clustering and cell type annotation of the cell cycle regressed oAL UMAP with clustering resolution set to 0.25. (G) Heatmap of top differentially expressed genes by cluster.

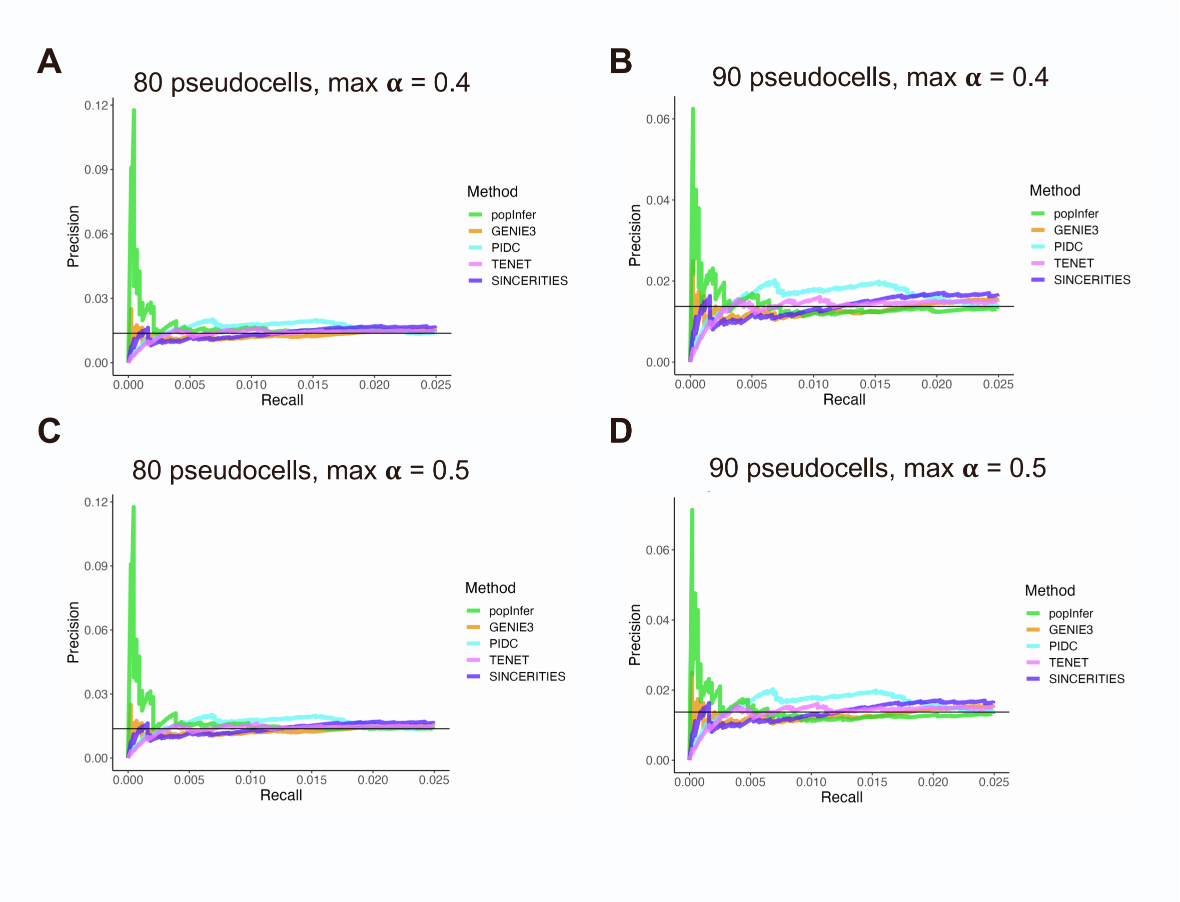

Figure S2: Early precision-recall curves for the HSC to GMP transition in oAL for different numbers of pseudocells and α sequences: (A) 80 pseudocells, $\alpha \in \{0, 0.001, 0.002, \ldots, 0.4\}$, (B) 90 pseudocells, $\alpha \in \{0, 0.001, 0.002, \ldots, 0.4\}$, (C) 80 pseudocells, $\alpha \in \{0, 0.001, 0.002, \ldots, 0.5\}$, (D) 90 pseudocells, $\alpha \in \{0, 0.001, 0.002, \ldots, 0.5\}$.

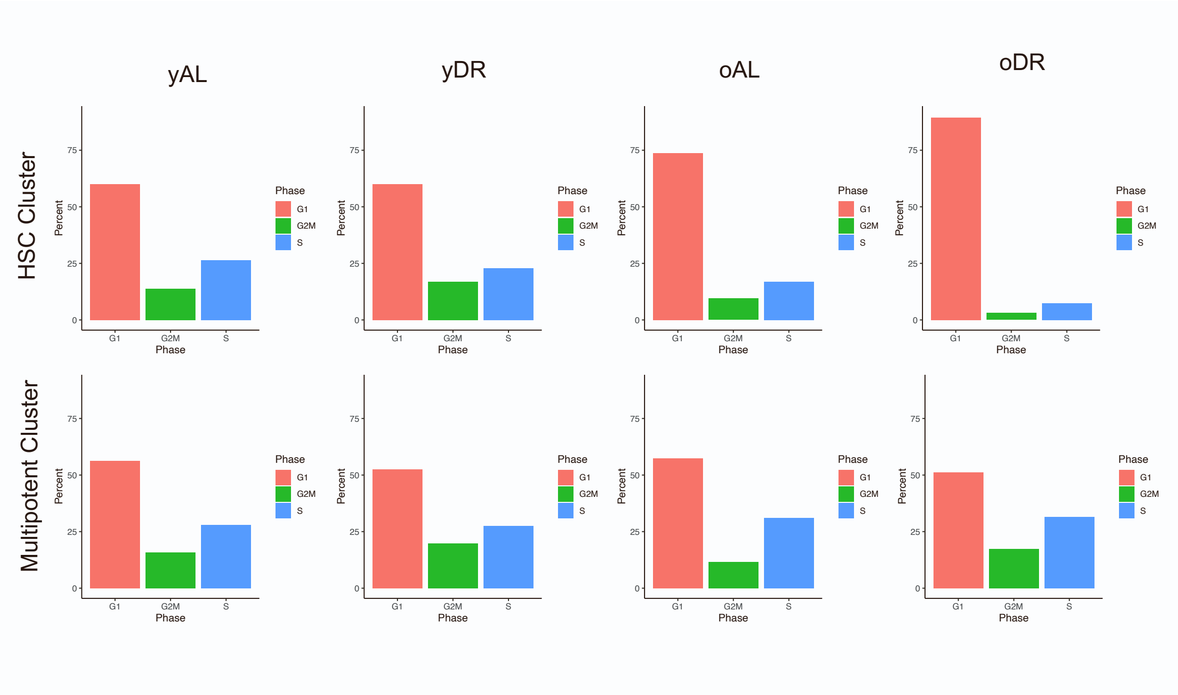

Figure S3: Percent of HSC and multipotent cells in each cell cycle phase by sample.

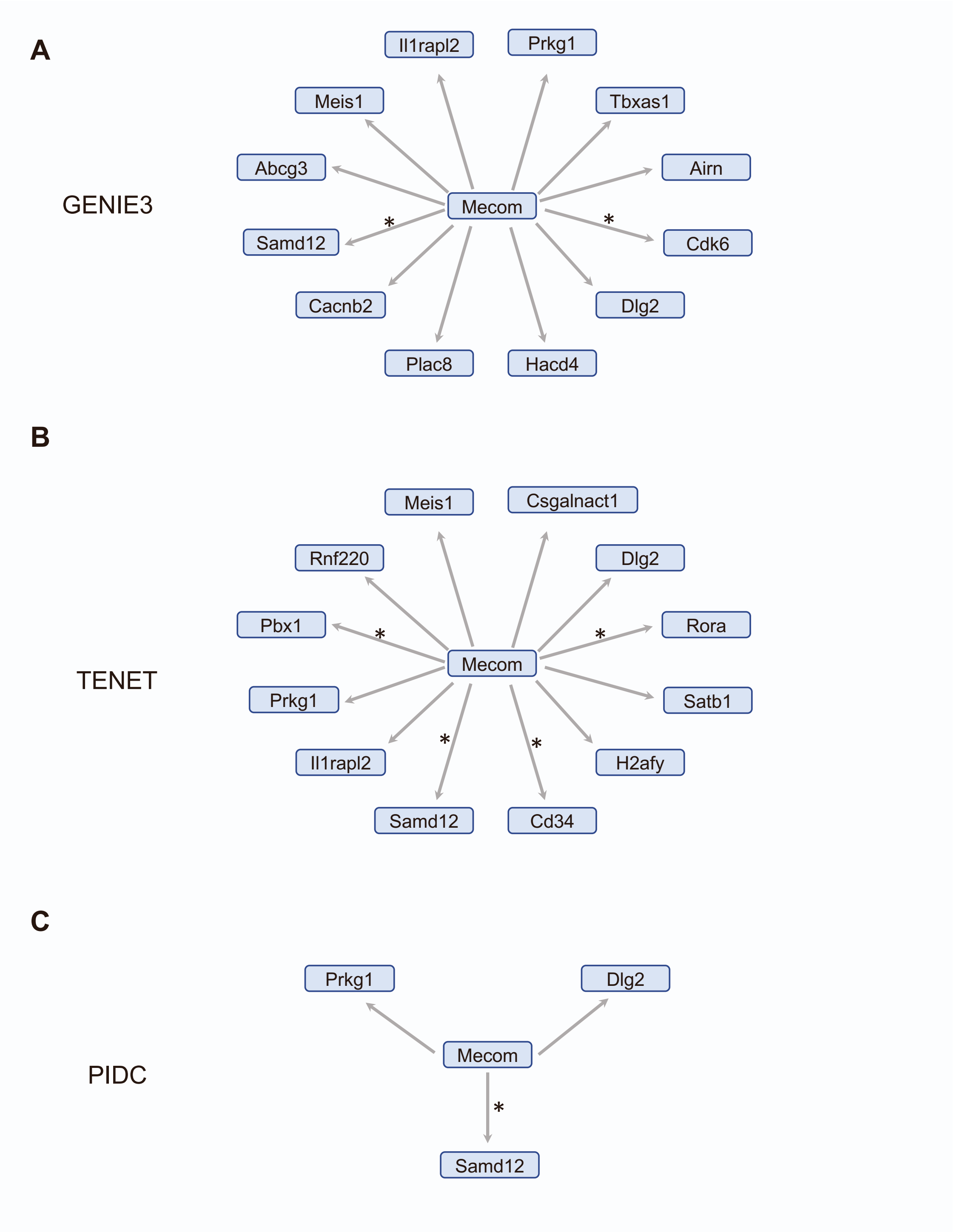

Figure S4: Subnetworks of yAL *Mecom* targets inferred by (A) GENIE3, (B) TENET, and (C) PIDC. Edges marked with black stars correspond to target genes that were differentially expressed upon *Evi1* (encoded by *Mecom*) activation.

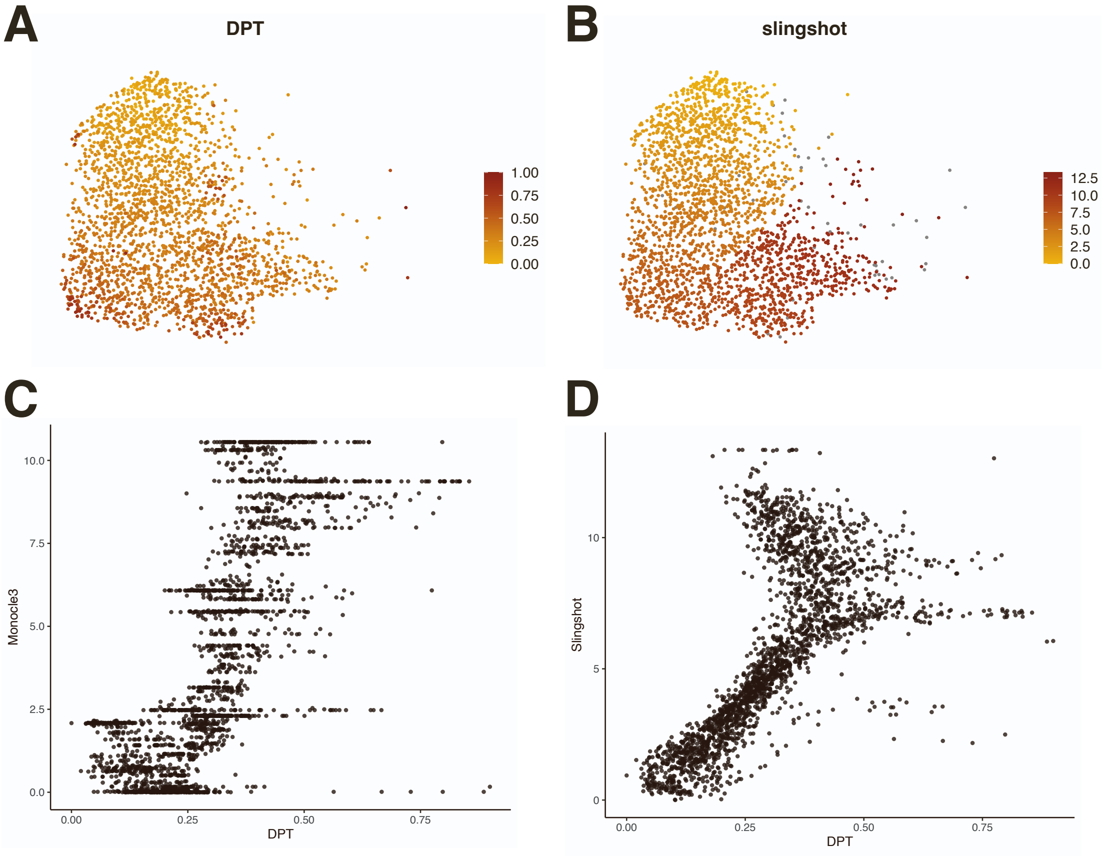

Figure S5: Comparison of algorithms for pseudotime. (A) DPT pseudotime projected onto HSCs and MPPs in yAL. (B) Slingshot pseudotime projected onto HSCs and MPPs in yAL. (C) Correlation between DPT and Monocle pseudotimes. (D) Correlation between DPT and Slingshot pseudotimes.

Supplementary Tables

Fluorescent antibodies used for cell staining

Reagent	Company	Catalogue Number	Clone Number
Biotin TER 119	Bio Legend	116204	TER-119
Biotin GR1	Bio Legend	108404	RB6-8C5
Biotin CD11b	Bio Legend	101204	M1/70
Biotin B220	Bio Legend	103204	RA3-6B2
Biotin CD4	Bio Legend	100508	RM4-5
Biotin CD8a	Bio Legend	100704	53-6.7

Table SI 1: Mature cell fluorescence AB mix to stain lineage-markers.

Reagent	Company	Catalogue Number	Clone Number
CD150 Brilliantviolet 605	Bio Legend	115927	TC15-12F12.2
c-kit APC	Bio Legend	105812	2B8
SCA1 (Ly-6A/E) PE	Bio Legend	108108	D7
Streptavidin APC-Cy7	Bio Legend	405208	-
CD34 FITC	Invitrogen	11-0341-85	RAM34
CD16/32 PE-Cy7	Bio Legend	101318	93

Table SI 2: HSPC fluorescence AB mix.

Cell type	Marker/ definition
LSK	Lin-/c-kit+/Sca1+
MEP	Lin-/c-kit+/Sca1-/FcR-/CD34-
CMP	Lin-/c-kit+/Sca1-/FcR-/CD34+
GMP	Lin-/c-kit+/Sca1-/FcR+/CD34+
MPP	Lin-/c-kit+/Sca1+/CD34+
HSC	Lin-/c-kit+/Sca1+/CD34-/ CD150+

Table SI 3: Markers of HSPC populations

	Stock	Final	Volume for 1ml in μl
20x Nuclei Buffer	20x	1x	50
DTT	1000mM	1mM	1
RNase inhibitor	40U/μl	1U/μl	25
Nuclease free water	-	-	924

Table SI 4: Diluted nuclei buffer

	Stock	Final	Volume for 4ml in μl
Tris-HCl pH 7.4	1M	10mM	40
NaCl	5M	10mM	8
MgCl2	1M	3mM	12
BSA	10%	1%	400
Tween-20	10%	0.1%	40
DTT	1000mM	1mM	4
RNase inhibitor	40U/μl	1U/μl	100
Nuclease free water	-	-	3400

Table SI 5: Wash buffer

	Stock	Final	Volume for 2ml in μl
Tris-HCl pH 7.4	1M	10mM	20
NaCl	5M	10mM	4
MgCl2	1M	3mM	6
Tween-20	10%	0.1%	20

Table SI 6: Lysis buffer
